# Combinatorial Viral Vector-Based and Live Attenuated Vaccines without an Adjuvant to Generate Broader Immune Responses to Effectively Combat Pneumonic Plague

**DOI:** 10.1128/mBio.03223-21

**Published:** 2021-12-07

**Authors:** Paul B. Kilgore, Jian Sha, Emily K. Hendrix, Vladimir L. Motin, Ashok K. Chopra

**Affiliations:** a Department of Microbiology & Immunology, University of Texas Medical Branch, Galveston, Texas, USA; b Institute for Human Infections and Immunity, University of Texas Medical Branch, Galveston, Texas, USA; c Department of Pathology, University of Texas Medical Branch, Galveston, Texas, USA; d Sealy Institute for Vaccine Sciences, University of Texas Medical Branch, Galveston, Texas, USA; e Galveston National Laboratory, University of Texas Medical Branch, Galveston, Texas, USA; University of Texas Southwestern Medical Center Dallas

**Keywords:** humoral, mucosal, cell-mediated immune responses, capsular antigen F1-minus mutant, heterologous prime-boost or simultaneous immunization, Rag1 knockout mice, *Yersinia pestis* CO92, pneumonic plague mouse model

## Abstract

Mice immunized with a combination of an adenovirus vector (Ad5-YFV) and live-attenuated (LMA)-based vaccines were evaluated for protective efficacy against pneumonic plague. While the Ad5-YFV vaccine harbors a fusion cassette of three genes encoding YscF, F1, and LcrV, LMA represents a mutant of parental Yersinia pestis CO92 deleted for genes encoding Lpp, MsbB, and Ail. Ad5-YFV and LMA were either administered simultaneously (1-dose regimen) or 21 days apart in various orders and route of administration combinations (2-dose regimen). The 2-dose regimen induced robust immune responses to provide full protection to animals against parental CO92 and its isogenic F1 deletion mutant (CAF^−^) challenges during both short- and long-term studies. Mice intranasally (i.n.) immunized with Ad5-YFV first followed by LMA (i.n. or intramuscularly [i.m.]) had higher T- and B-cell proliferative responses and LcrV antibody titers than those in mice vaccinated with LMA (i.n. or i.m.) first ahead of Ad5-YFV (i.n.) during the long-term study. Specifically, the needle- and adjuvant-free vaccine combination (i.n.) is ideal for use in plague regions of endemicity. Conversely, with a 1-dose regimen, mice vaccinated with Ad5-YFV i.n. and LMA by the i.m. route provided complete protection to animals against CO92 and its CAF^−^ mutant challenges and elicited Th1/Th2, as well as Th17 responses, making it suitable for emergency vaccination during a plague outbreak or bioterrorist attack. This is a first study in which a viral vector-based and live-attenuated vaccines were effectively used in combination, representing adjuvant- and/or needle-free immunization, with each vaccine triggering a distinct cellular immune response.

## INTRODUCTION

The zoonotic bacterium Yersinia pestis, the causative agent of bubonic and pneumonic plague, is a Tier-1 select agent. Historically, three plague pandemics resulted in more than 200 million deaths worldwide (1). The endemic nature of the disease has resulted in an ever-increasing number of human plague cases worldwide, including the United States, with a case fatality rate of 18% (2). In the most recent major outbreak of plague in Madagascar (2017), although millions of doses of levofloxacin were prescribed to treat people suspected of contracting plague, the mortality rate still was at 8.6% (3). Notably, in this outbreak, >75% of the cases were pneumonic in nature, in contrast to traditional bubonic plague, the latter of which has a slower disease course and a longer window for effective antibiotic treatment (4, 5). However, for pneumonic plague, antibiotic therapy must be initiated within 24 h of the onset of symptoms to be effective, and, if left untreated, fatality rate is almost 100% (6). Therefore, vaccination is the most effective way to prevent plague. Unfortunately, there are no Food and Drug Administration (FDA)-approved plague vaccines (7), and those currently under clinical trials are all based on two antigens: F1 (capsular antigen) and LcrV (low-calcium response V antigen, a type 3 secretion system [T3SS] component) (8–10). Consequently, in individuals vaccinated with the F1-V-based vaccines and infected with the F1-negative strain of Y. pestis, protection would be reliant on only immune responses generated against LcrV. Indeed, an LcrV-based vaccine alone did not provide complete protection against pneumonic plague caused by F1-negative Y. pestis challenge, and the F1-V-based vaccines were much less effective against bubonic and pneumonic plagues upon challenge with the F1-negative Y. pestis strain C12 (11–13). Similarly, the F1-V-based vaccines were not completely protective against pneumonic plague in African green monkeys (14). More alarming is the existence of LcrV polymorphism in Y. pestis as well as variants in Y. enterocolitica and Y. pseudotuberculosis strains with little cross protection (15–18). Therefore, it is necessary to develop new plague vaccines and evaluate different immunization strategies that could be effective against the F1-negative Y. pestis strains, those which harbor LcrV variants, as well as against human population with various immune responses in regions of endemicity or during an outbreak/biothreat situation.

In our laboratory, we have developed two types of plague vaccine candidates, namely, LMA and Ad5-YFV. The live attenuated vaccine LMA is a triple deletion mutant of Y. pestis CO92 in which genes encoding Braun lipoprotein (Lpp), an acyltransferase (MsbB), and the attachment invasion locus (Ail) were deleted (19). While Lpp activates proinflammatory cascade by binding to toll-like receptor 2 (TLR-2) (20, 21), MsbB adds lauric acid to the lipid A moiety of lipopolysaccharide (LPS), which triggers TLR-4 signaling (22, 23). Ail, in addition to promoting attachment and invasion of Y. pestis to the host, provides serum resistance to the organism (19, 24).

Ad5-YFV is a replication-deficient adenovirus type 5 vector-based vaccine containing genes for three plague antigens: fraction 1 capsule-like antigen F1, tip protein of the T3SS LcrV, and YscF, which forms the barrel structure of the T3SS needle (25). Using a prime-boost immunization regimen (21 days apart), both vaccines, when used individually, elicited robust humoral and cell-mediated immune responses in animals and conferred 100% protection against lethal Y. pestis CO92 challenge (26, 27). Importantly, each of these vaccines has its own unique characteristics. Notably, we observed a significant induction of CD4^+^ IL-17^+^-producing T cells in LMA vaccine-immunized mice (27, 28). IL-17 production is an important correlate of protection against plague in the absence of protective antibodies (29, 30). However, such a T-cell population was not detected in animals immunized with the Ad5-YFV vaccine (26). Further, the Th1 immune response was favored after vaccination of mice with the Ad5-YFV vaccine over Th2, possibly due to the Ad5 vector used, while the Th2 immune response was favored after immunization of animals with the LMA vaccine (26, 28). In our past studies, the Ad5-YFV vaccine was always delivered intranasally (i.n.), while the LMA vaccine was administered by the i.m. or the i.n. route (19, 25–28).

Like other live attenuated vaccines, one of the major advantages of LMA is that it delivers a large array of antigens in their native state, closely mimicking natural infection, and such vaccines are expected to provide protection against all circulating Y. pestis variants in nature (30–34). While the viral vector-based vaccines, especially the replication-deficient ones such as Ad5-YFV, are much safer than the live attenuated vaccines in general, both types of vaccines have their own disadvantages. The most obvious limitation of live attenuated vaccines is the safety profile, especially in immunocompromised individuals, and the potential concern for reversion (35–37). However, the LMA mutant was rationally designed with complete deletion of three genes located at different locations on the bacterial genome (19). Because of its high level of virulence attenuation and rapid clearance (within 12 to 24 h) while retaining immunogenicity in animals (27), LMA vaccine was excluded from the Centers for Disease Control and Prevention (CDC) select agent list (https://www.selectagents.gov/sat/exclusions/hhs.html).

The Ad5-YFV vaccine only incorporates three plague antigens, F1, LcrV, and YscF, and the F1-minus strains of Y. pestis have been isolated from humans/animals and are as virulent as the wild-type (WT) plague bacterium (38, 39). In addition, hypervariable regions within the LcrV protein have been described, and antibody responses to these LcrV variants are not cross-protective (18). Therefore, we have successfully added a third protective antigen, YscF, in this vaccine to circumvent disadvantages of F1-V-based vaccines (25, 40). However, it is still plausible that the Ad5-YFV vaccine alone is not efficacious against all circulating Y. pestis variants, although was shown to be protective (100%) against the CAF^−^ mutant of CO92 (26). Importantly, neither of our vaccines requires an adjuvant to boost immune responses, unlike F1-V-based vaccines that employ alum (8, 9, 11, 41–43).

To alleviate some of the above-described concerns, in this study, we carried out a heterologous immunization strategy in which both LMA and Ad5-YFV vaccines were administered either simultaneously (1-dose regimen) or in a prime-boost format (2-dose regimen). Our results showed almost all the heterologous vaccination groups of mice induced robust immune responses and provided full protection against lethal challenge doses of both parental and CAF^−^ mutant strains of Y. pestis CO92, albeit using potentially slightly different mechanisms. This is the first detailed plague vaccine study with heterologous vaccination regimens that involved a live attenuated vaccine LMA and a viral vector-based vaccine, Ad5-YFV.

## RESULTS

### Virulence and immunogenic characterization of the LMA vaccine candidate in iron-overloaded conventional or immunocompromised mice.

We have previously shown the LMA mutant to be highly attenuated in conventional (immunocompetent) mice (19, 27). To further evaluate its attenuation, we tested the safety of the LMA mutant during iron overload conditions in conventional mice to mimic hemochromatosis. We also tested its safety in immunocompromised Rag1 knockout (KO) mice.

**FIG 1:**
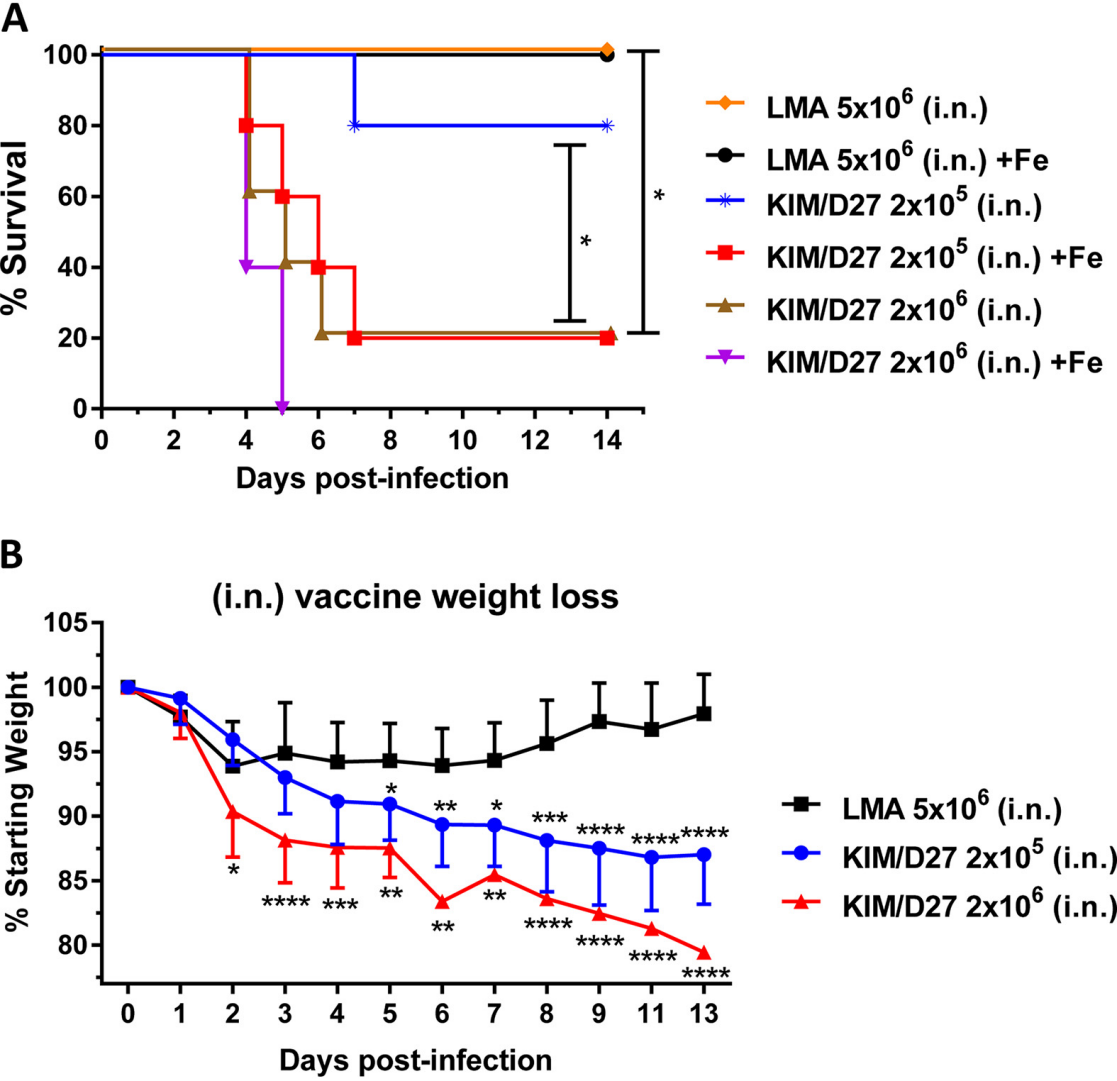
Iron overload condition does not restore virulence of the LMA vaccine. (A and B) Swiss-Webster mice (*n* = 5/group) were injected with 75 μg of FeCL_2_·4H_2_O 1 h prior to infection and then infected with either the LMA vaccine or the KIM/D27 strain. Losses in body weight and mortality were monitored for 14 days; animals without iron overload were used as controls. Kaplan-Meier analysis with log-rank (Mantel-Cox) test was used for analysis of animal survival. Two-way ANOVA with Tukey’s *post hoc* test was used to calculate significant differences in body weight loss between vaccine groups. Asterisks represent the statistical significance between two groups, indicated by a line for panel A and between LMA and the indicated dose of KIM/D27 in panel B. Since absence or presence of iron did not affect animal body weight, combined data were plotted. Two biological replicates were performed. *, *P* < 0.05; **, *P* < 0.01; ***, *P* < 0.001; ****, *P* < 0.0001.

We demonstrated that all of the mice challenged with the LMA vaccine (5 × 10^6^ CFU, more than double the vaccination dose) by the i.n. route survived irrespective of whether the animals were iron overloaded or not (Fig. 1A), with a minimal loss in body weight (Fig. 1B). On the contrary, mice infected with 2 × 10^6^ CFU of the KIM/D27 strain died by day 5 in the presence of FeCl_2_, while 80% of the animals succumbed to infection without iron overload (Fig. 1A). At a lower challenge dose of 2 × 10^5^ CFU, 80% of the iron-overloaded mice succumbed by day 7, while only 20% of the non-iron-overloaded mice succumbed (Fig. 1A). In contrast to the LMA mutant, KIM/D27 strain-infected mice showed a dramatic loss in body weight even at the lower challenge dose of 2 × 10^5^ CFU (Fig. 1B).

We then infected Rag1 KO mice with 4 50% lethal doses (LD_50_) of CO92 by either the i.n. or the i.m. route, since these two routes were previously used for vaccination studies with LMA (19, 27, 28). As shown in Fig. S1A in the supplemental material, 100% of mice infected by the i.n. or the i.m. route succumbed to infection by day 4 to 5 postinfection (p.i.), with up to ∼20% body weight loss by day 4. We then examined bacterial burden in the lungs, liver, and spleen of these mice and found greater than 10^8^ CFU of Y. pestis in both the lungs and the spleen, while a significantly higher Y. pestis burden, with up to more than 10^9^ CFU, was observed in the liver (Fig. S1B).

10.1128/mBio.03223-21.1 FIG S1 Parental Y. pestis CO92 causes clinical disease in Rag1 KO mice. (A) C57BL6 Rag1 KO mice (*n* = 4 to 5 each route) were infected with 4 LD_50_ of CO92 by either i.n. or i.m. route and the mortality of animals recorded and plotted. (B) Lungs, liver, and spleen were removed from the moribund animals to quantify the bacterial loads. (A) Kaplan-Meier analysis with log-rank (Mantel-Cox) test was used for analysis of animal survival. (B) Asterisks represent the statistical significance between two groups indicated by a line. ***, *P* < 0.001. Two biological replicates were performed and data plotted. Download FIG S1, PDF file, 0.1 MB. Copyright © 2021 Kilgore et al. 2021 Kilgore et al. https://creativecommons.org/licenses/by/4.0/ This content is distributed under the terms of the Creative Commons Attribution 4.0 International license.

**FIG 2:**
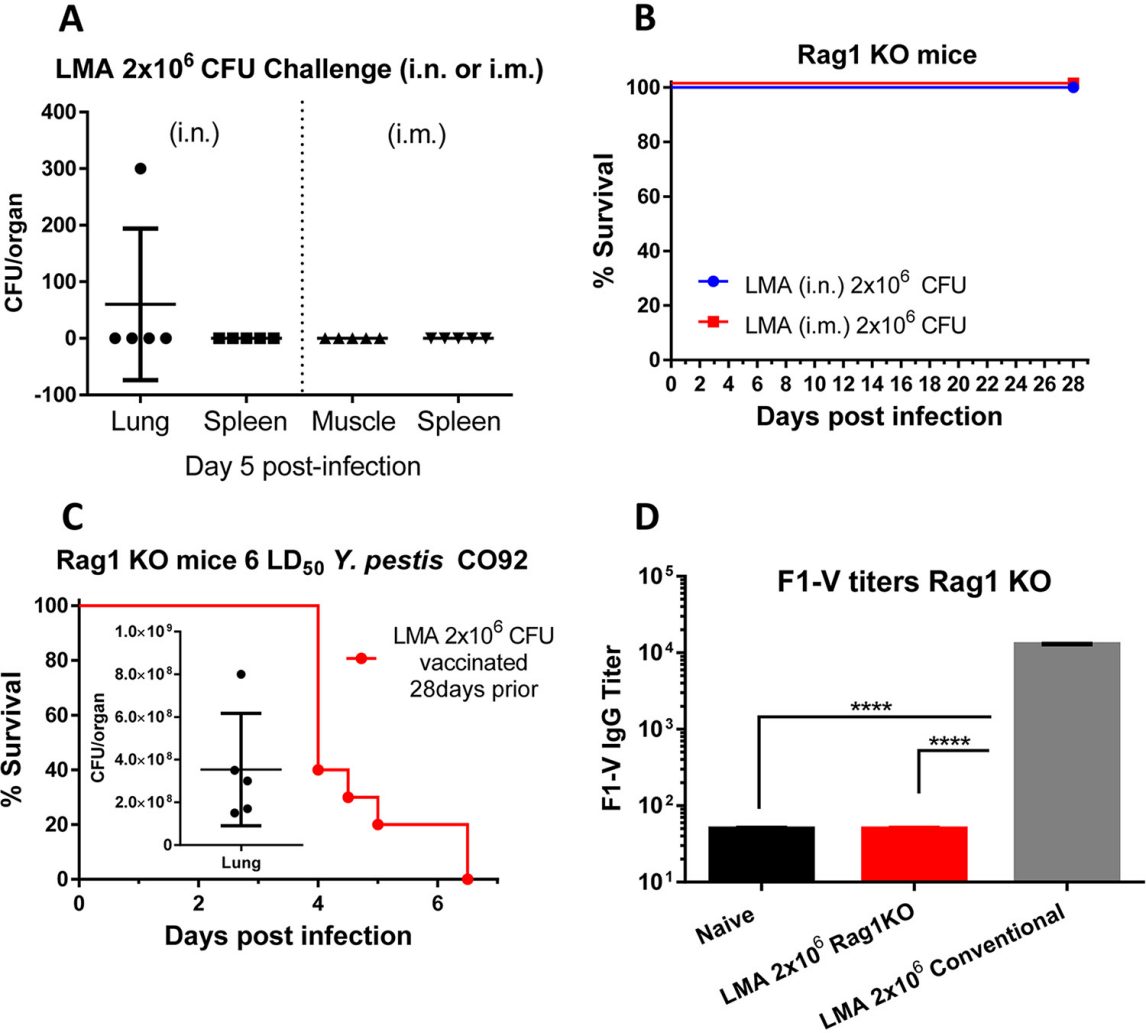
Attenuation and immunologic characterization of LMA vaccine in Rag1 KO mice. C57BL6 Rag1 KO mice were infected with 2.0 × 10^6^ CFU of LMA by either the i.n. or the i.m. route (*n* = 10 each route). (A) On day 5 p.i., 5 animals from each infection route were euthanized, and spleen, lungs, or muscle (depending on the infection route) was collected to quantify the number of LMA. (B) The survival of the remaining LMA infected animals (5 from each route) was monitored for up to 28 days p.i. After 28 days of LMA infection, all surviving mice (*n* = 10) were challenged with 6 LD_50_ of CO92. (C) The mortality of animals was recorded, and bacterial loads in the lungs from 5 moribund animals (inset) were enumerated. F1-V-specific IgG titers were evaluated by ELISA from sera collected at day 3 post-CO92 challenge. Sera collected from uninfected naive Rag1 KO mice and conventional mice immunized with LMA (2.0 × 10^6^ CFU i.m.) served as negative and positive controls, respectively (D). One-way ANOVA was used to determine significance between groups for bacterial burdens and antibody titers. Kaplan-Meier analysis with log-rank (Mantel-Cox) test was used for analysis of animal survival. Asterisks represent the statistical significance between two groups, indicated by a line. ***, *P* < 0.001. Two biological replicates were performed and data plotted.

We then evaluated the LMA mutant, when delivered at a vaccination dose of 2.0 × 10^6^ CFU by both i.n. and i.m. routes, in Rag1 KO mice. Five days p.i., five mice infected with LMA from each infection route were necropsied and examined for the presence of the mutant either at the infection site (lungs or muscle) or in the spleen as the result of systematic dissemination. As shown in Fig. 2A, there was no detectable LMA mutant in the spleen of mice infected by either the i.n. or the i.m. route. We also did not detect any LMA mutant at the injection site of muscle. We did enumerate 300 CFU in the lungs of one mouse that was infected by the i.n. route; however, this number was much lower than the initial infection dose of 2.0 × 10^6^ CFU. Further, no LMA mutant was detected in the lungs of other mice infected by the i.n. route (Fig. 2A). This was also indicated by the fact that all the mice infected with 2.0 × 10^6^ CFU of LMA survived up to 28 days p.i. (Fig. 2B) without any clinical signs of the disease and a minimal body weight loss similar to that shown in conventional mice (Fig. 1B).

These surviving mice were then challenged with 6 LD_50_ of CO92. As expected, all LMA-infected Rag1 KO mice succumbed to CO92 challenge with an overall >10^8^ CFU of Y. pestis CO92 present in the lungs (Fig. 2C) with ∼15% loss in body weight over time. Further, there were no F1-V-specific IgG antibodies in the sera of LMA (pooled from i.n.- and i.m.-infected) Rag1 KO mice on day 3 post-CO92 challenge compared to naive Rag1 KO mice. However, a significantly higher level of F1-V IgG antibodies was generated in the LMA-immunized conventional (immunocompetent) mice (pooled sera from i.n. and i.m. infection) from a parallel independent study (Fig. 2D).

### Strong immune responses were elicited in conventional mice by heterologous vaccination with either a 1- or 2-dose (prime-boost) regimen.

**FIG 3:**
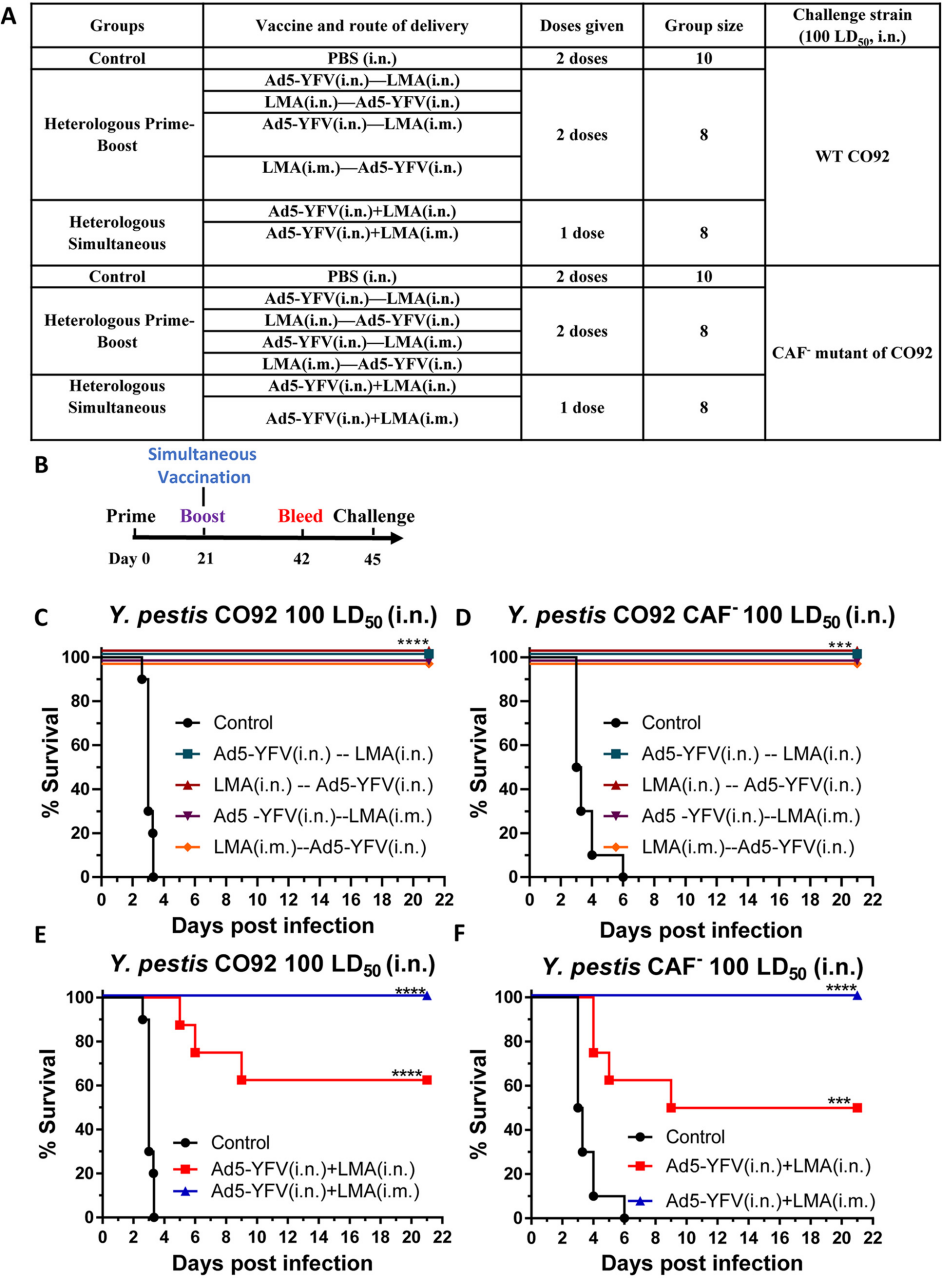
Short-term heterologous vaccination study with conventional mice. Mice (*n* = 8 to 10 per group) were immunized heterologously with Ad5-YFV and LMA vaccines in either a 1- or a 2-dose regimen. In the 1-dose regimen, both Ad5-YFV and LMA vaccines were delivered simultaneously, while in a 2-dose regimen, Ad5-YFV and LMA vaccines were administered 21 days apart in various orders and combinations. Mice receiving PBS were used as controls. The composition of the groups and the experiment time course are depicted in panels A and B, respectively. Three weeks after completion of the vaccination schedule, mice were challenged with 100 LD_50_ of either CO92 (C and E) or its F1 deletion mutant, CAF^−^ (D and F), and observed for morbidity and mortality for 21 days. Kaplan-Meier analysis with log-rank (Mantel-Cox) test was used for analysis of animal survival. Asterisks represent the statistical significance between the indicated groups to the naive control mice. ***, *P* < 0.001; ****, *P* < 0.0001. Two biological replicates were performed and data plotted.

Mice were immunized with Ad5-YFV and LMA vaccines in either a 1- or 2-dose regimen. In a 1-dose regimen, both Ad5-YFV and LMA vaccines were administered simultaneously, while in a 2-dose regimen, Ad5-YFV and LMA vaccines were delivered 21 days apart in various order and route combinations (Fig. 3A). The immunization schedule for either 1- or 2-dose regimens is depicted in Fig. 3B. Three weeks after completion of the vaccinations, the immunized and control mice were challenged with 100 LD_50_ of either CO92 or its F1 deletion mutant, CAF^−^. As shown in Fig. 3C and D, all of the 2-dose heterologous prime-boost vaccinated mice, regardless of the order in which the vaccines were administered or the route of vaccination by which LMA was delivered, were 100% protected from both CO92 and CAF^−^ strain challenge with no body weight loss and other clinical symptoms of the disease. A level of 100% protection was also observed for mice simultaneously immunized with Ad5-YFV (i.n.) and LMA (i.m.) during CO92 and CAF^−^ challenges. However, when both Ad5-YFV and LMA vaccines were simultaneously administered i.n., the immunized mice had 50% (during CAF^−^ strain challenge) and 63% (during CO92 challenge) survival rates (Fig. 3E and F).

**FIG 4:**
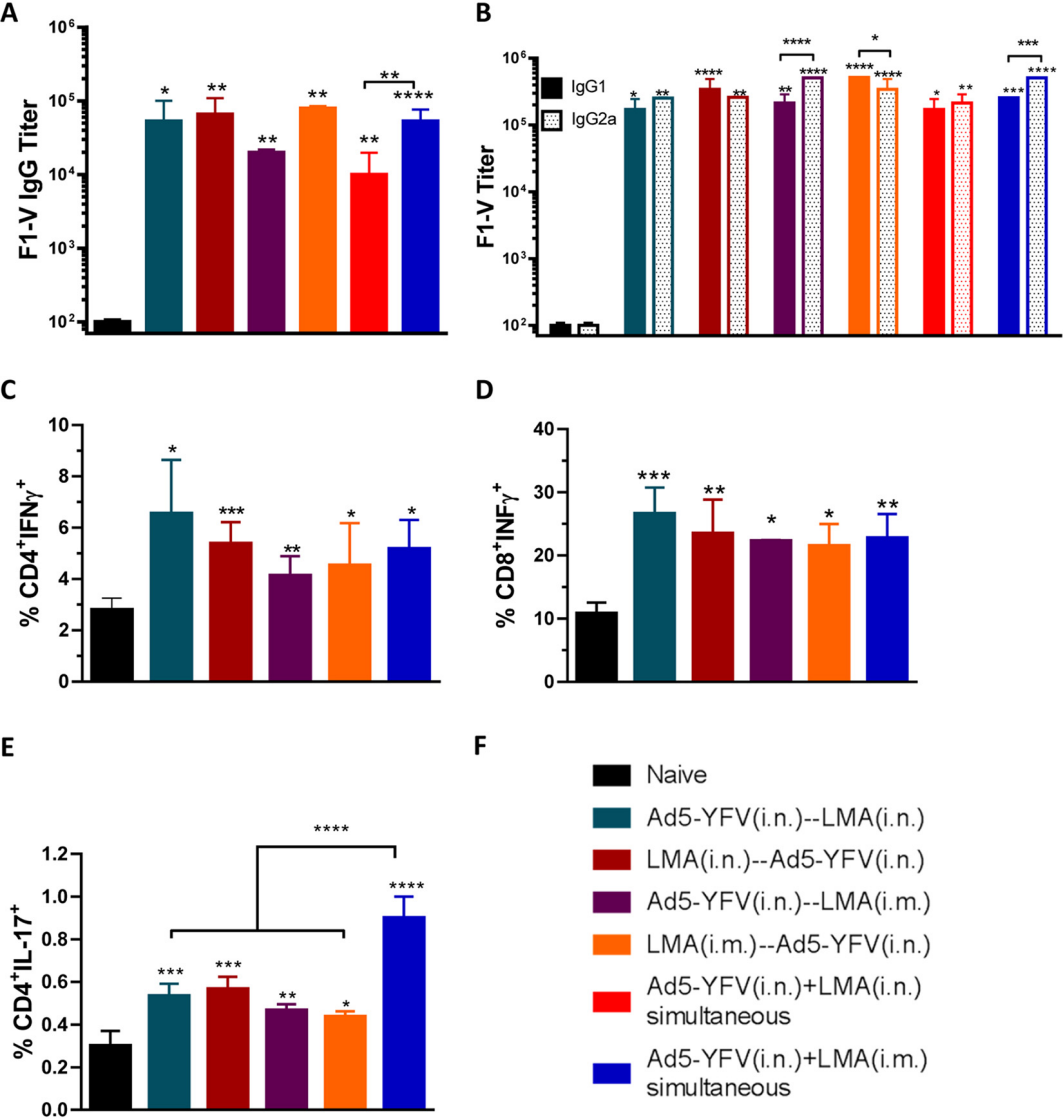
Humoral and cell-mediated immune responses during short-term heterologous vaccination study. Sera were collected 21 days after the last immunization from both immunized and naive control mice as described for Fig. 3. ELISA was performed to evaluate specific F1-V total IgG titers (A) as well as its isotype IgG1 and IgG2a titers (B). In a separate experiment, mice (*n* = 5) were similarly immunized as described for Fig. 3; however, the group in which mice were simultaneously immunized with LMA and Ad5-YFV i.n. was excluded. Twenty-one days after completion of the vaccination course, spleens were harvested. Splenocytes were isolated and stimulated with PMA, ionomycin, and brefeldin A. Subsequently, splenocytes were surface stained for CD3, CD4, and CD8, followed by intracellular staining for IFN-γ (C and D) and IL-17A (E). Different groups of mice used are depicted in panel F. Statistical analysis was performed using one-way ANOVA with Tukey’s *post hoc* test (A, C, D, and E) or two-way ANOVA with Tukey’s *post hoc* test (B) to determine significance. Asterisks directly above bars indicated significance compared to control group, while asterisks with comparison bars denoted significance between the indicated groups. *, *P* < 0.05; **, *P* < 0.01; ***, *P* < 0.001; ****, *P* < 0.0001. Two biological replicates were performed and data plotted. *In vitro* studies had 3 replicates.

We then measured IgG antibody titers to rF1-V fusion protein in sera of immunized mice collected on day 42 prior to the challenges. In general, all vaccinated groups of animals had notable increases in antibody titers, which were 2 to 3 logs higher than that of the naive controls (Fig. 4A). Among the immunized mice, relatively lower F1-V antibody titers were noted in animals immunized with either a 2-dose regimen of Ad5-YFV (i.n.)-LMA (i.m) or a 1-dose regimen of Ad5-YFV (i.n.) plus LMA (i.n.). However, a significant difference was only observed between the 2 simultaneously immunized groups of mice (Fig. 4A, red versus blue bars).

We also examined the isotypes of F1-V IgG antibodies to gauge Th1 versus Th2 bias. In general, all vaccinated groups of mice had significantly higher levels of F1-V-specific IgG1 and IgG2a than the naive animals. For simultaneously vaccinated groups of mice, there were generally higher levels of F1-V-specific IgG2a over IgG1; however, a significant difference was only observed for animals that received Ad5-YFV (i.n.) and LMA (i.m.) (Fig. 4B). For the 2-dose-vaccinated groups of mice, when Ad5-YFV was administered first (teal and purple bars), there were always higher levels of IgG2a over IgG1, while the difference was significant only in the Ad5-YFV (i.n.)-LMA (i.m.)-immunized group of mice, as shown in purple bars (Fig. 4B). In contrast, when the LMA vaccine was delivered as the first dose (crimson and orange bars), higher levels of IgG1 over IgG2a were observed, but again these differences were only significant when LMA was administered by the i.m. route, as shown in orange bars (Fig. 4B).

We next examined cell-mediated immune responses in immunized mice from the groups that showed 100% protection during the Y. pestis challenges (Fig. 3C to F). In a separate experiment, splenocytes were isolated from vaccinated mice 21 days after the last vaccination dose and stimulated with phorbol 12-myristate 13-acetate (PMA) and ionomycin. All vaccination groups had significantly higher populations of CD4^+^ gamma interferon-positive (IFN-γ^+^), CD8^+^ IFN-γ^+^, and CD4^+^ interleukin-17-positive (IL-17^+^) cells than those of naive mice (Fig. 4C to E). Among the vaccinated groups, mice i.n. immunized with either LMA or Ad5-YFV first in a 2-dose regimen (teal and crimson bars) showed slightly higher percentages of IFN-γ-producing CD4^+^ and CD8^+^ T cells than all other groups of immunized mice; however, no significant differences were observed (Fig. 4C and D).

On the other hand, notably higher populations of IL-17-producing CD4^+^ T cells were noticed in the group of mice simultaneously immunized with Ad5-YFV (i.n.) and LMA (i.m.) compared to all the 2-dose-regimen-immunized groups of mice (Fig. 4E, blue bar). Interestingly, although it did not reach a significant level, mice i.n. immunized with either LMA or Ad5-YFV first in a 2-dose regimen (teal and crimson bars) showed slightly higher percentages of IL-17-producing CD4^+^ T cells than all other 2-dose-regimen-immunized groups of animals. A trend similar to that of IL-17 was also observed for the IFN-γ-producing CD4^+^ and CD8^+^ T cells (Fig. 4C and D).

### Robust immune response was sustained in conventional mice with a 2-dose regimen vaccination during a long-term study.

**FIG 5:**
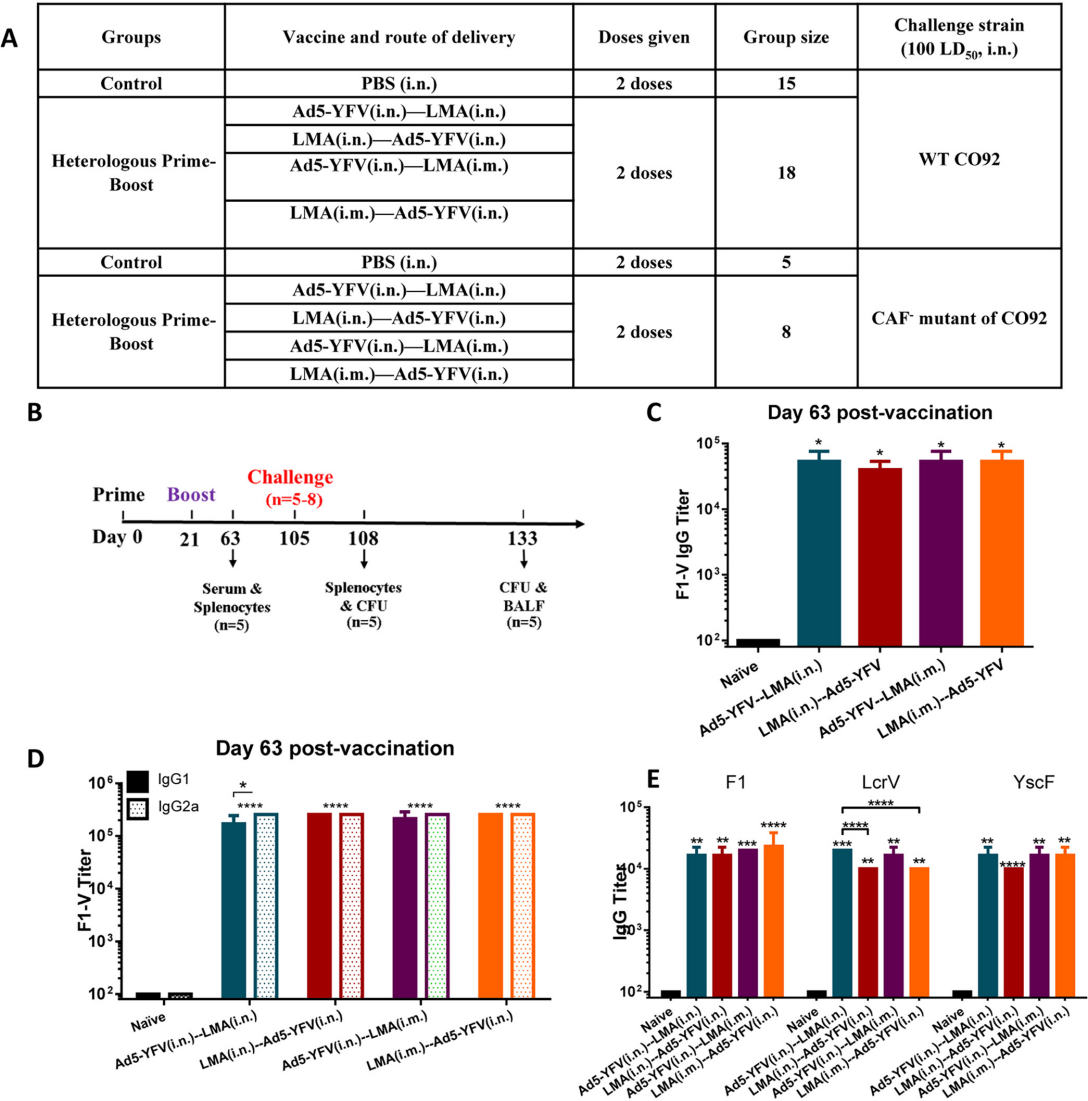
Antibody responses during long-term heterologous prime-boost vaccination study. Mice were immunized with Ad5-YFV and LMA vaccines in 2-dose (prime-boost) regimens in which Ad5-YFV and LMA were administered 21 days apart in various combinations. The composition of the groups and the experiment time course are depicted in panels A and B, respectively. Sera were collected on day 63 of the study, which was 42 days after the 2nd vaccination dose. The total IgG and its isotype IgG1/IgG2a titers specific to F1-V were determined by ELISA and are displayed in panels C and D, respectively, while the total IgG titers specific to individual antigens F1, LcrV, and YscF are shown in panel E. Statistical significance was determined by one-way ANOVA with Tukey’s *post hoc* test (A, C, and D) and by two-way ANOVA with Tukey’s *post hoc* test (B). Asterisks directly above bars indicated significance compared to the control group, while asterisks with comparison bars denoted significance between the indicated groups. *, *P* < 0.05; **, *P* < 0.01; ***, *P* < 0.001; ****, *P* < 0.0001. Two biological replicates were performed and data plotted. *In vitro* studies had 3 replicates.

After examining the above-described survival data and immune responses from both 1- and 2-dose heterologous vaccination regimens, we chose to focus on the 2-dose regimen for further evaluation in a long-term vaccination study. The sole reason for this was to develop a needle-free vaccination protocol, although comparisons were also made with the i.m.-vaccinated animals. During the long-term study, we examined humoral and cell-mediated responses at 42 days after the 2nd dose of vaccination as well as at 3 days post-CO92 challenge. The details of immunization regimens and schedules of the study are shown in Fig. 5A and B.

As shown in Fig. 5C, similar levels of F1-V-specific antibodies were detected in the sera across all vaccinated groups of mice and were all significantly higher (>2 logs) than that of naive control animals (Fig. 5C). Further, antibody isotype analysis revealed a generally balanced level of IgG1 and IgG2a in the immunized mice except for animals immunized first with Ad5-YFV (teal and purple bars), which had higher levels of IgG2a over IgG1. However, the difference was only significant in mice immunized with Ad5-YFV (i.n.)-LMA (i.n.) (Fig. 5D, teal bars).

To further dissect the antibodies elicited by the vaccination, we evaluated antibody titers to each individual plague antigen F1, LcrV, and YscF, which were the components of the Ad5-YFV vaccine. As shown in Fig. 5E, all immunized mice produced a similar level of IgG against each individual antigen, and they were all significantly higher than that of naive control mice. To be more specific, antibody titers against F1 and YscF were comparable among all vaccinated groups of mice. However, antibody titers against LcrV were significantly higher in mice immunized with Ad5-YFV (i.n.)-LMA (i.n.) (teal bar) than mice vaccinated with either LMA (i.n.)-Ad5-YFV (i.n.) (crimson bar) or LMA (i.m.)-Ad5-YFV (i.n.) (orange bar) (Fig. 5E).

**FIG 6:**
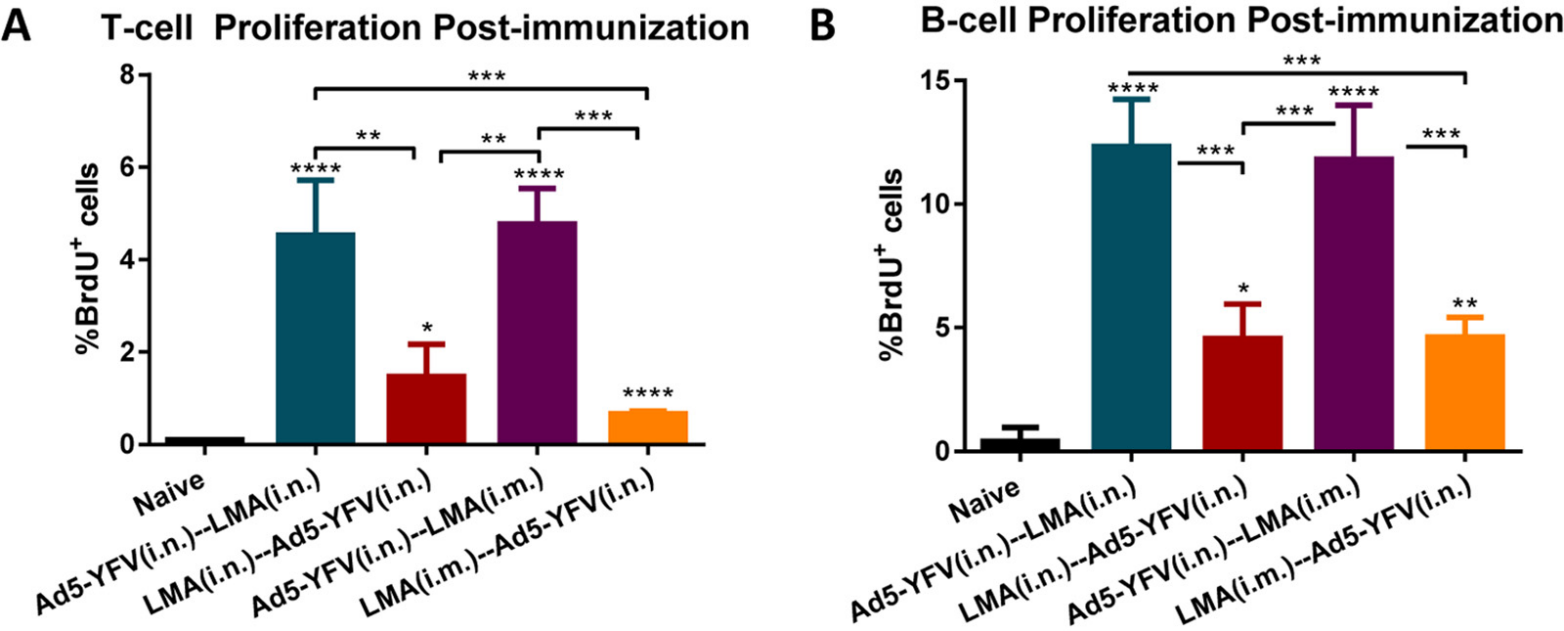
T- and B-cell proliferation in response to heterologous prime-boost vaccination during long-term study. Spleens were collected 21 days after the last vaccination dose from a cohort (*n* = 5 per group) of immunized and naive control mice as described for Fig. 5. The isolated splenocytes were stimulated with rF1-V (100 μg/ml) for 72 h and 37°C, and then BrdU was added at a final concentration of 10 μM during the last 18 h of incubation with rF1-V to be incorporated into newly synthesized DNA of the splenocytes. Subsequently, the BrdU-labeled splenocytes were surface stained for T- and B-cell markers followed by BrdU and 7-AAD staining. The splenocytes were then subjected to flow cytometry, and the percentage of BrdU-positive cells in CD3 (A)- and CD19 (B)-positive populations were calculated using FACSDiva software. Statistical significance was determined using one-way ANOVA with Tukey’s *post hoc* test. Asterisks above columns represented comparison to the control group, while asterisks with comparison bars denoted significance between the indicated groups. *, *P* < 0.05; **, *P* < 0.01; ***, *P* < 0.001; ****, *P* < 0.0001. Two biological replicates were performed and data plotted. *In vitro* studies had 3 replicates.

The isolated splenocytes from mice 42 days post-2nd dose of vaccine were stimulated with Y. pestis-specific rF1-V fusion antigen (100 μg/ml) to induce cell proliferation by measuring incorporation of bromodeoxyuridine (BrdU) in the newly synthesized chromosomal DNA (44). As shown in Fig. 6, significant T- and B-cell proliferation was generally noticed in all of the vaccinated groups compared to the naive controls. Interestingly, a much stronger T- and B-cell proliferation was achieved in mice that were first vaccinated with Ad5-YFV (teal and purple bars) than those mice that were first immunized with LMA (crimson and orange bars) regardless of the route by which LMA was administered (Fig. 6). It is plausible that administration of LMA first might have somewhat of a toxic effect on T and B cells, an effect not observed when Ad5-YFV was delivered first and could account for less T- and B-cell proliferation. It is important that antibody titers to LcrV (Fig. 5E) were also lower when LMA was used first followed by Ad5-YFV for vaccination of mice. We suspect that antibodies to YscF, the third component in the Ad5-YFV vaccine, compensate for this lower LcrV antibody titer in protection.

### Conventional mice vaccinated with the 2-dose regimen were fully protected from CO92 and CAF^−^ challenges during a long-term study.

**FIG 7:**
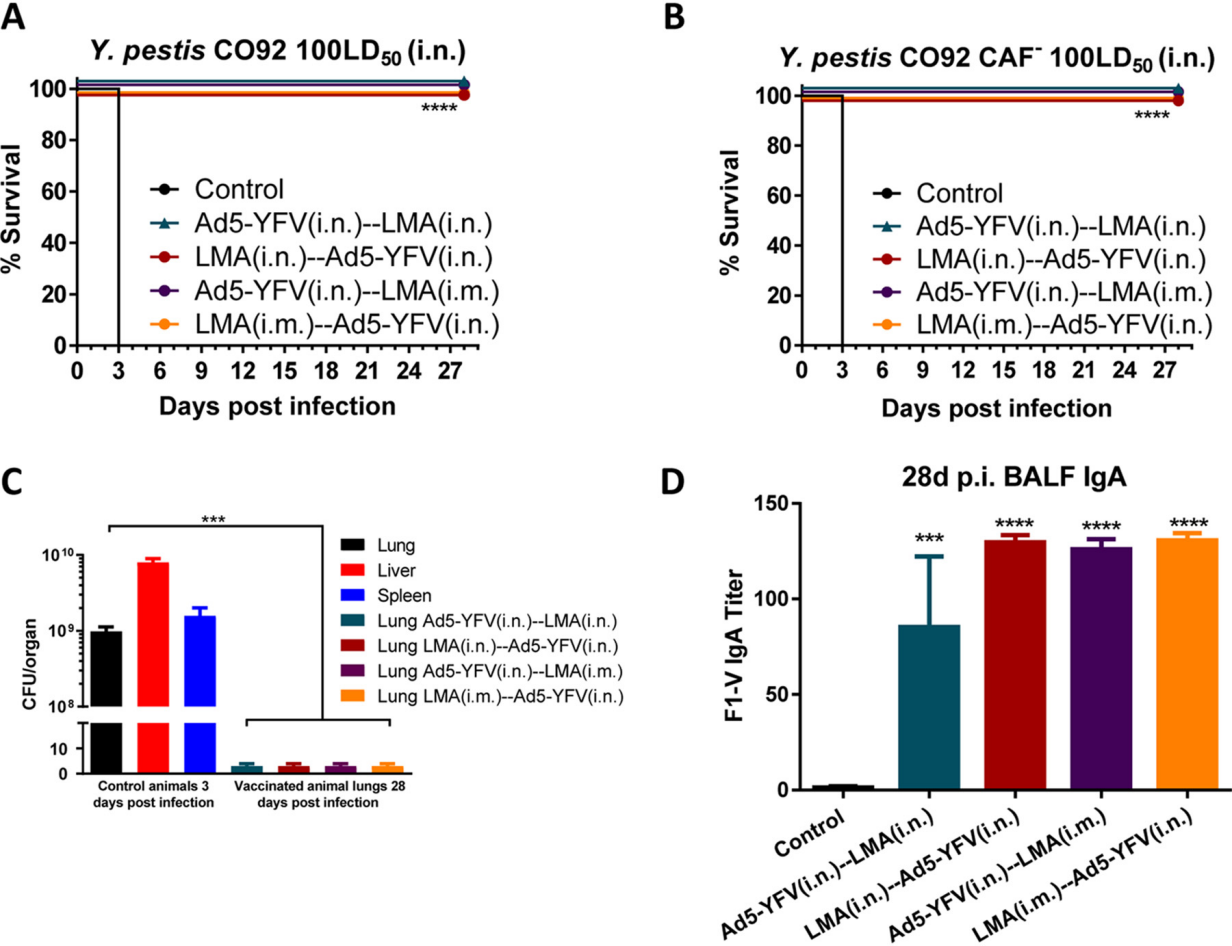
Heterologous prime-boost vaccinations provide protection to immunized mice in long-term study. A cohort (*n* = 5 to 8 per group) of immunized and naive control mice as described for Fig. 5 were challenged on day 105 of the study (84 days after 2nd vaccination) with 100 LD_50_ of either CO92 (A) or its CAF^−^ strain (B) and monitored for morbidity and mortality for 28 days. (C) On day 3 p.i., lungs, liver, and spleen were excised from all moribund mice to quantify bacterial load. At the end of the study (on day 28), BALFs and lungs were collected from the terminated animals. Lung homogenates were plated to determine the clearance of Y. pestis from the surviving animals. (D) The collected BALFs were evaluated for IgA titers specific to F1-V by ELISA, and PBS-injected mice were used as controls. One-way ANOVA was used to determine significance between groups for bacterial burdens and antibody titers, while Kaplan-Meier analysis with log-rank (Mantel-Cox) test was used for analysis of animal survival. Asterisks represented the statistical significance compared to the control group or between the two groups, indicated by a line. ***, *P* < 0.001; ****, *P* < 0.0001. Two biological replicates were performed and data plotted. *In vitro* studies had 3 replicates.

The immunized mice along with naive controls were then i.n. challenged with 100 LD_50_ of either CO92 or its CAF^−^ strain. As expected, all mice in vaccinated groups survived (with no clinical symptoms of the disease), while 100% of naive control animals succumbed to infection (Fig. 7A and B, with up to 20% body weight loss). In addition, high levels of plague bacilli (10^9^ to 10^10^ CFU/organ) were detected in various organs of diseased naive control mice. In contrast, the inoculated Y. pestis was completely cleared from infected lungs of all the vaccinated mice after 28 days of challenge (Fig. 7C). We have previously shown that mice vaccinated with either 2 doses of LMA or Ad5-YFV cleared the invading pathogen within 3 days p.i. (26, 27). At the end of the study (day 28 postchallenge), we collected bronchoalveolar fluid (BALF) from all surviving mice, and all vaccinated and challenged animals had a significant level of F1-V-specific IgA compared to phosphate-buffered saline (PBS) controls (Fig. 7D).

We then examined sera of mice for IgA postvaccination and postchallenge with CO92, and no differences in titers were noted (Fig. S2), suggesting infection did not further enhance serum IgA levels. A similar study will be performed in the future assessing IgA levels in BALF postvaccination and postchallenge. Mice from the Ad5-YFV (i.n.)-LMA (i.n.)-vaccinated group had slightly lower levels of IgA than the other heterologous prime-boost-vaccinated groups of animals, although the differences were not significant (Fig. 7D). Although it is expected that in pneumonic plague IgA would significantly contribute to host protection, some studies indicated a minimal protective role of IgA (31, 45). However, whether the immune status of the host could be a contributing factor in IgA-associated protection is unclear and needs further investigation.

10.1128/mBio.03223-21.2 FIG S2 No significant differences in F1-V-specific serum IgA are observed in vaccinated mice before and after infection. Mice were immunized with Ad5-YFV and LMA vaccines in 2-dose (prime-boost) regimens in which Ad5-YFV and LMA were administered 21 days apart in various combinations (Fig. 5A). Serum was collected 42 days after the 2nd vaccination as well as 28 days postinfection. F1-V specific IgA was determined by ELISA. Titers were determined in triplicate. One-way ANOVA with Tukey’s *post hoc* test was used to determine significant differences between groups. Asterisks indicate significance compared to naïve serum. ****, *P* < 0.0001. Download FIG S2, PDF file, 0.09 MB. Copyright © 2021 Kilgore et al. 2021 Kilgore et al. https://creativecommons.org/licenses/by/4.0/ This content is distributed under the terms of the Creative Commons Attribution 4.0 International license.

**FIG 8:**
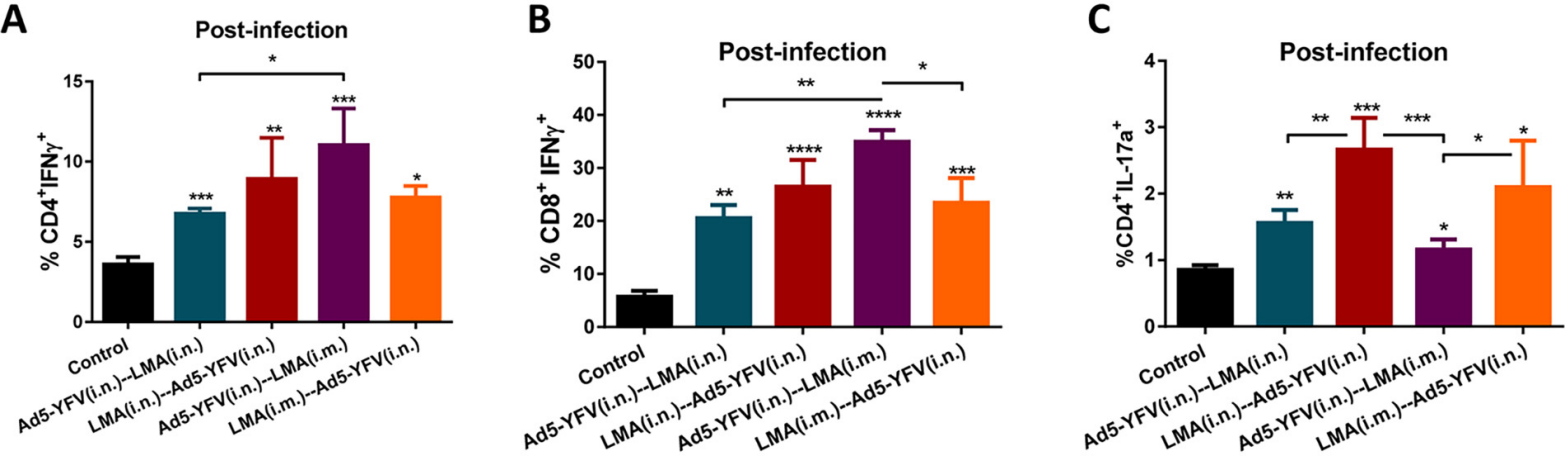
T-cell responses to CO92 challenge during long-term heterologous prime-boost vaccination study. A cohort (*n* = 5 per group) of immunized and naive control mice as described for Fig. 5 was challenged on day 105 of the study (84 days after 2nd vaccination) with 100 LD_50_ of CO92. Spleens were harvested on day 3 p.i., and the isolated splenocytes were then stimulated with PMA, ionomycin, and brefeldin A. Cells were stained with T-cell surface markers CD3, CD4, and CD8, followed by intracellular IFN-γ and IL-17A staining. Percentages of CD4^+^ IFN-γ^+^ (A), CD8^+^ IFN-γ^+^ (B), and CD4^+^ IL-17^+^ cells (C) are shown. Cells were then analyzed by flow cytometry. Statistical significance was determined using one-way ANOVA with Tukey’s *post hoc* test as well as Student's *t* test. Asterisks above columns represented comparison to the control group, while asterisks with comparison bars denoted significance between other indicated groups. *, *P* < 0.05; **, *P* < 0.01; ***, *P* < 0.001; ****, *P* < 0.0001. Two biological replicates were performed and data plotted. *In vitro* studies had 3 replicates.

To gauge cell-mediated immunity of vaccinated mice in response to CO92 challenge, on day 3 p.i., splenocytes were isolated from both immunized and control animals and subjected to flow analysis after PMA-ionomycin stimulation. We noted statistically higher IFN-γ-producing CD4^+^ and CD8^+^ T- cell populations across all of the vaccinated mice compared to that of naive control animals (Fig. 8A and B), and a similar trend was observed for the IL-17-producing CD4^+^ T cells (Fig. 8C).

More specifically, a significantly higher level of CD4^+^ IFN-γ^+^ population was observed for mice immunized (purple bar) with Ad5-YFV (i.n.)-LMA (i.m.) than for mice vaccinated (teal bar) with Ad5-YFV (i.n.)-LMA (i.n.) (Fig. 8A). On the other hand, a significant difference in CD8^+^ IFN-γ^+^ population was only noticed between Ad5-YFV (i.n.)-LMA (i.m.)-immunized (purple bar) compared to Ad5-YFV (i.n.)-LMA (i.n.) (teal bar)- or LMA (i.m.)-Ad5-YFV (i.n.) (crimson bar)-vaccinated groups of mice (Fig. 8B).

In terms of CD4^+^ IL-17^+^ population, mice immunized first with LMA either by the i.n. or the i.m. route generally revealed better levels than mice immunized with Ad5-YFV vaccine first. Significant differences were observed between LMA (i.n.)-Ad5-YFV (i.n.)-immunized group (crimson bar) of mice and groups vaccinated with either Ad5-YFV (i.n.)-LMA (i.n.) (teal bar) or Ad5-YFV (i.n.)-LMA (i.m.) (purple bar) as well as between groups immunized with LMA (i.m.)-Ad5-YFV (i.n.) (orange bar) and the group vaccinated with Ad5-YFV (i.n.)-LMA (i.m.) (purple bar) (Fig. 8C).

### Characterization of mice splenic cytokine and chemokine profiles in response to vaccination and CO92 challenge.

**FIG 9:**
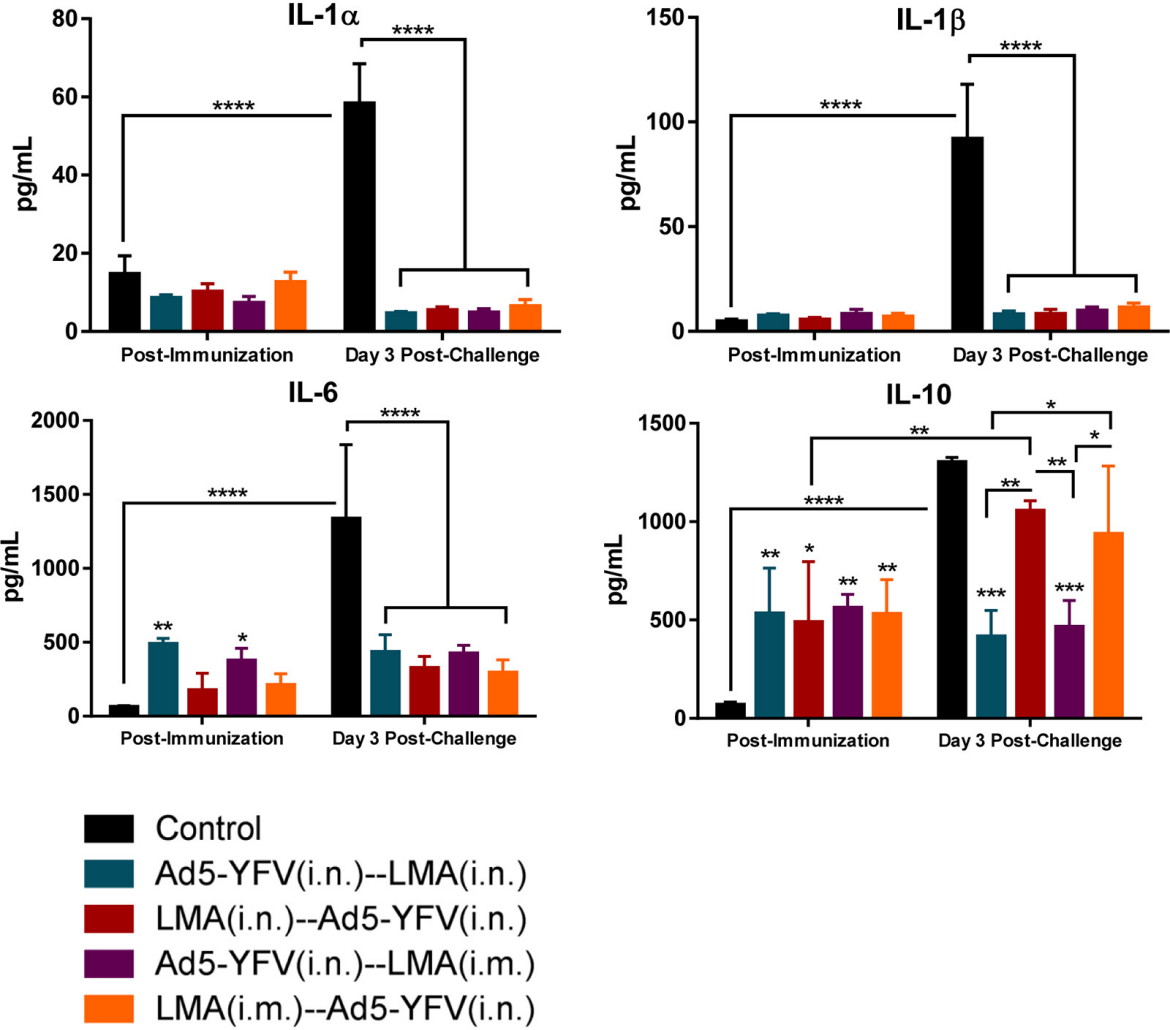
Splenocyte proinflammatory and anti-proinflammatory responses during long-term heterologous prime-boost vaccination study. The splenocytes isolated from mice described for Fig. 6 (postimmunization) and Fig. 8 (post-CO92 challenge) were further stimulated with rF1-V (100 μg/ml) for 3 days. The cytokines in the culture supernatants were analyzed by using Bioplex-23 assay and expressed as the arithmetic means ± standard deviations. The proinflammatory and anti-proinflammatory cytokines are shown. Statistical significance was determined using two-way ANOVA with Tukey’s *post hoc* test to compare multiple time points or Student's *t* test to compare 2 groups within the same time point. Asterisks above columns represented comparison to the control group, while horizontal bars represented differences between test groups. *, *P* < 0.05; **, *P* < 0.01; ***, *P* < 0.001; ****, *P* < 0.0001. Two biological replicates were performed and data plotted. *In vitro* studies had 3 replicates.

To further evaluate cell-mediated immunity of mice in response to vaccination and infection, splenocytes collected either postimmunization or postchallenge (Fig. 5B) were stimulated with rF1-V to examine cytokine/chemokine production. We divided up the cytokine/chemokine analysis into 3 panels: proinflammatory/anti-proinflammatory cytokines, Th1/Th2/Th17-associated cytokines, and chemokines. At postvaccination time points, the overall proinflammatory cytokines in immunized mice were either slightly elevated (IL-6) or remained at levels (IL-1α and IL-1β) comparable to those of naive control mice (Fig. 9). However, in response to Y. pestis infection, significantly increased proinflammatory cytokines were observed only in naive control mice, while the levels of these cytokines were low and almost unchanged in vaccinated animals (Fig. 9). For the anti-proinflammatory cytokine IL-10, a significant increase was generally observed in all of the immunized mice compared to that of naive controls at the postimmunization time point. In response to Y. pestis infection, the level of IL-10 was significantly elevated in both naive control mice and in animals immunized first with LMA (crimson and orange bars); however, it remained unchanged in mice immunized first with Ad5-YFV (teal and purple bars) and was significantly lower than that of the naive control at the postchallenge time point (Fig. 9).

**FIG 10:**
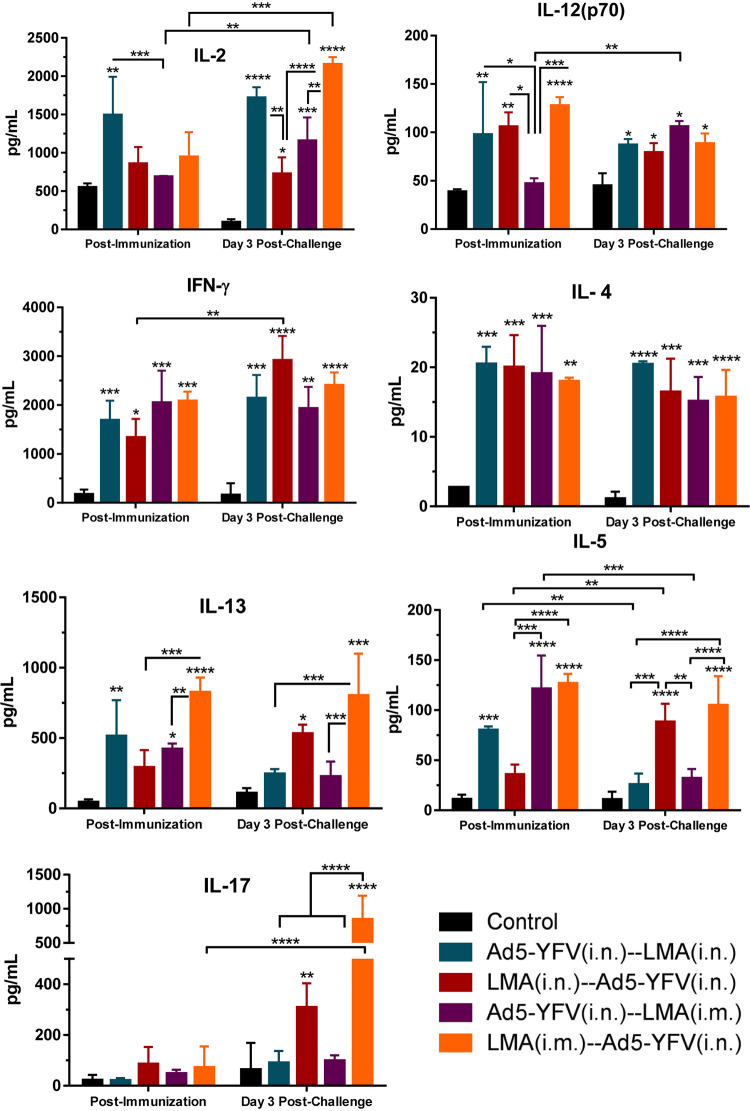
Splenocyte Th1/Th2/Th17 cytokine responses during long-term heterologous prime-boost vaccination study. Cytokine analysis was performed as described for Fig. 9. Th1/Th2/Th17 cytokine responses are shown. Statistical significance was determined using two-way ANOVA with Tukey’s *post hoc* test to compare multiple time points or Student's *t* test to compare 2 groups within the same time point. Asterisks above columns represented comparison to the control group, while horizontal bars represented differences between test groups. *, *P* < 0.05; **, *P* < 0.01; ***, *P* < 0.001; ****, *P* < 0.0001. Two biological replicates were performed and data plotted. *In vitro* studies had 3 replicates.

In contrast to proinflammatory cytokines, the Th1- and Th2-related cytokines such as IL-2, IL-12(p70), IFN-γ, IL-4, IL-5, and IL-13 were generally increased in all immunized mice compared to that of naive controls at the postimmunization time point (Fig. 10). On the other hand, IL-2, IL-12(p70), IFN-γ, and IL-4 levels were sustained at higher levels in all immunized groups in response to CO92 challenge. The levels of IL-5 and IL-13 were only maintained higher in mice first immunized with LMA (crimson and orange bars) but subsided to the level of naive controls in mice first vaccinated with Ad5-YFV (teal and purple bars) at the postchallenge time point (Fig. 10). The decline of Th2 cytokines IL-5 and IL-13 in mice immunized first with Ad5-YFV at the postchallenge time point might reflect its Th1 bias, as also shown above in analysis of IgG isotyping (Fig. 4B and 5D).

For Th17 response, slight increases in the levels of IL-17 were only observed in mice immunized first with LMA (crimson and orange bars) compared to that of the naive controls at postimmunization. In addition, the IL-17 level in these mice was further elevated in response to Y. pestis challenge. However, the IL-17 levels were consistently maintained at a low level and were comparable with that of naive controls at both time points (postimmunization and postchallenge) in mice vaccinated first with Ad5-YFV (teal and purple bars) (Fig. 10). It should be mentioned that it is difficult to precisely correlate percentage of T cells positive for IL-17 as assessed by flow cytometry and IL-17 secreted by these cells based on Bioplex, as the cells were stimulated with different agents, namely, PMA or F1-V; however, a correlative trend should be expected, as shown in Fig. 8C and 10.

**FIG 11:**
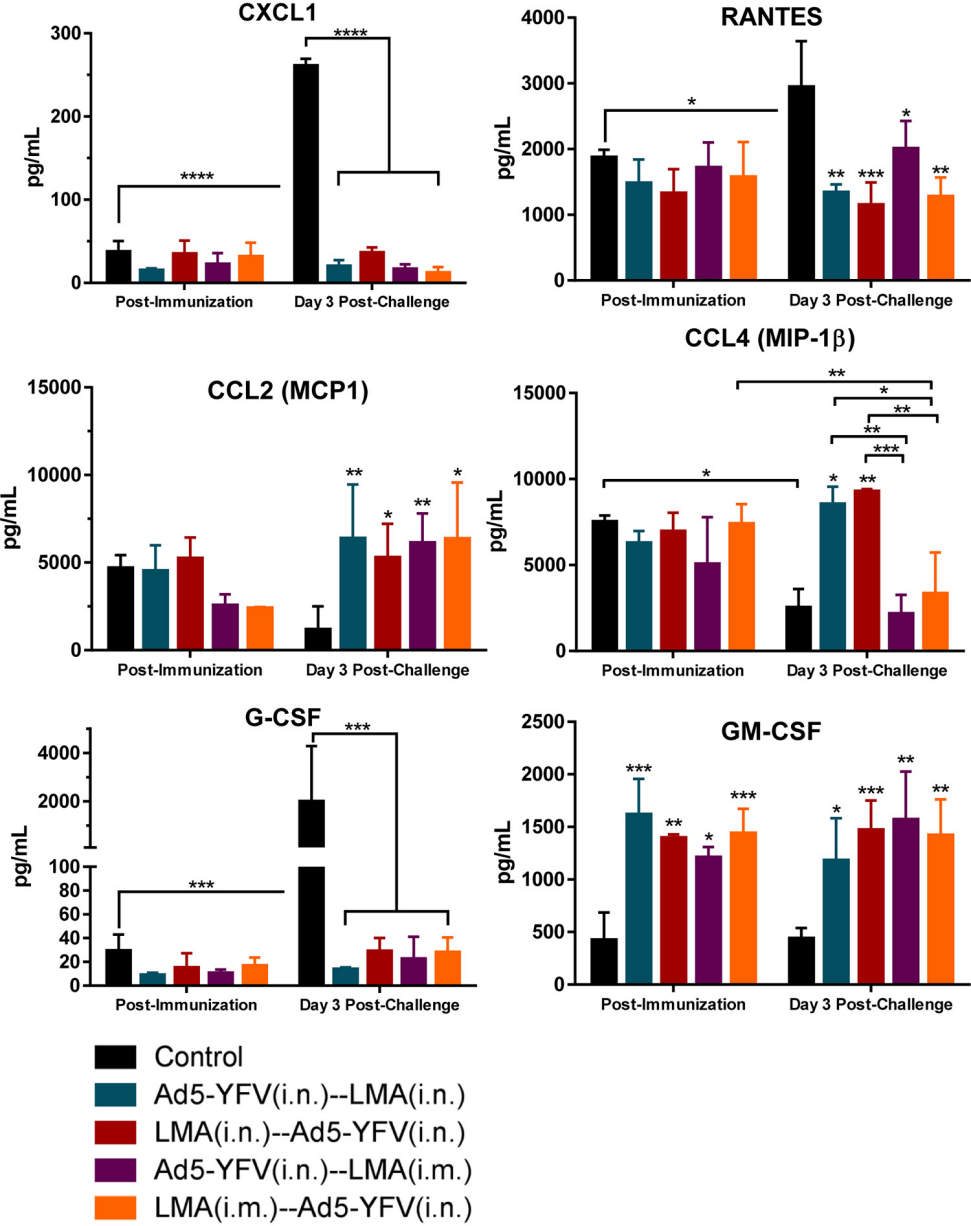
Splenocyte chemokine responses during long-term heterologous prime-boost vaccination study. Chemokine analysis was performed as described for Fig. 9. Chemokine responses are shown. Statistical significance was determined using two-way ANOVA with Tukey’s *post hoc* test to compare multiple time points or Student's *t* test to compare 2 groups within the same time point. Asterisks above columns represented comparison to the control group, while horizontal bars represented differences between test groups. *, *P* < 0.05; **, *P* < 0.01; ***, *P* < 0.001; ****, *P* < 0.0001. Two biological replicates were performed and data plotted. *In vitro* studies had 3 replicates.

In analysis of chemokine production, the overall levels of chemokines in all immunized mice were low and comparable with their corresponding naive controls at the postimmunization time point except for granulocyte-macrophage colony-stimulating factor (GM-CSF), which was significantly elevated (Fig. 11). In response to Y. pestis infection, CXCL1, RANTES, and G-CSF behaved similarly to proinflammatory chemokines and were significantly increased only in naive control mice but were at low levels and largely unchanged in all immunized and challenged animals (Fig. 11). In contrast, CCL2, CCL4, and GM-CSF had generally elevated levels in all immunized mice compared to that of the naive control at postchallenge time points, the exception being CCL4 in mice immunized with LMA i.m. (purple and orange bars), which was at a level to similar that of naive control challenged mice (Fig. 11).

## DISCUSSION

After the 2017 outbreak of plague in Madagascar, the WHO released a target product profile that outlined the desired characteristics for a potential plague vaccine (46). These characteristics included at most a 2-dose vaccination schedule, long-lasting protection with humoral and cell-mediated responses, possibility of a needle-free administration, and a robust safety profile, including potential use in pregnant women, children, and immunocompromised individuals (46).

We recently have developed two plague vaccine candidates: a live-attenuated vaccine, LMA, and an Ad5 viral vector-based vaccine, Ad5-YFV; individually both of them provided complete protection to immunized animals against challenge with CO92 (25–28). In response to the WHO requirements, here, we first further investigated the safety of the LMA vaccine in an iron overload condition and then in Rag1 KO mice (47). In live-attenuated plague strains that rely on pigmentation locus mutations such as KIM/D27 and EV76, hereditary hemochromatosis has been shown to restore bacterial virulence (48), while the LMA mutant has an intact pigmentation locus with functional T3 and T6 secretion systems (19 and data not shown).

Unlike the KIM/D27 strain, which exhibited more virulence in an iron overload environment, LMA vaccine was not affected by the presence of more iron in mice (Fig. 1). The *Rag1* gene defect in humans is associated with a broad spectrum of clinical and immunological phenotypes and is one of the major causes of human immune deficiency (PID) (49, 50). The Rag1 KO mice receiving up to 2.0 × 10^6^ CFU of LMA, which is equivalent to 20,000 LD_50_ of CO92, did not exhibit any clinical symptoms of the disease, and the LMA mutant rapidly cleared from these mice (Fig. 2). These data demonstrated a high degree of attenuation imparted by the selected mutations in LMA and provided an indication that the vaccine would be suitable for use in immunocompromised individuals.

We then implemented a heterologous immunization strategy in which both LMA and Ad5-YFV vaccines were delivered either simultaneously (1-dose regimen) or in a prime-boost format (2-dose regimen), as we hypothesized such a strategy would have several advantages. First, the use of two rationally designed vaccines, which are based on different principles, would complement each other from their own potential disadvantages, such as limited plague antigens in the Ad5-YFV vaccine versus the LMA vaccine, which would provide immune responses to a plethora of antigens. Second, the use of an Ad5-YFV vaccine as the first dose would negate any safety concerns of employing LMA as the second booster dose. Third, the heterologous immunization scheme is expected to mount unique and durable immune responses by integrating characteristics of each of the two vaccines, leading to superior protection in a broader human population. Fourth, the 1-dose regimen would shorten the immunization course and is considered ideal to be used in emergency situations such as a plague outbreak or during a bioterrorist attack.

Indeed, all our heterologous immunizations (irrespective of the order of vaccine delivery and the routes of administration) induced robust immune responses in mice and provided full protection to animals against the lethal challenges of both CO92 and its CAF^−^ mutant. The exception was the group of mice that received Ad5-YFV and LMA vaccines simultaneously by the i.n. route and had 50% to 63% survival rates during CO92 or its CAF^−^ strain challenges (Fig. 3). This relatively lesser protection rate in mice was correlated well with lower F1-V-specific antibody titers in mouse serum compared to animals simultaneously immunized with Ad5-YFV and LMA via different routes, i.e., i.n. and i.m., respectively (Fig. 4A). Further, there could be interference in triggering protective immune responses when both LMA and Ad5-YFV vaccines were delivered simultaneously in the lungs. It is also plausible that a stronger innate immunity developed in the lungs due to administration of two vaccines simultaneously, resulting in their respective rapid clearance and, thus, decreasing overall immunogenicity. Similar results were reported in a study in which the tuberculosis (TB) vaccine BCG was used in combination with other TB subunit vaccines (e.g., 85A, E6, and TB10.4) in a heterologous format. Simultaneous administration of them via different routes (pulmonary and parental) induced both pulmonary and systemic immunities, resulting in better protection than animals that were simultaneously vaccinated via the same route (51). Interestingly, the Ad5-YFV (i.n.) and LMA (i.m.) simultaneous vaccination combination also exhibited immune response characteristics of both Ad5-YFV (Th1) and LMA (Th17) vaccines (Fig. 4B and E) with complete protection of mice against challenges with CO92 and its CAF^−^ mutant (Fig. 3E and F). Thus, a 1-dose regimen of two vaccines provided us with an excellent tool to combat plague during emergency situations.

It is difficult to discern which ones of our 2-dose heterologous regimens were better, as all of them provided full protection to the immunized animals with comparable levels of F1-V-specific antibodies and overall similar cytokine profiles. However, an intriguing phenomenon emerged in that the immune profile of 2-dose immunization was likely dictated by the vaccine that was administered first.

More specifically, when the Ad5-YFV vaccine was delivered first, Th1 immune response was more pronounced based on F1-V-specific IgG2a/IgG1 antibody ratio (Fig. 4B) and the splenic cytokine profiles (Fig. 10). Likewise, when LMA vaccine was administered first followed by that of Ad5-YFV, the Th2 immune response was favored (Fig. 3B and 8C) along with that of Th17 (Fig. 8C and 10). Further, clear differences were noted in terms of T- and B-cell proliferation when mice were immunized first with the Ad5-YFV vaccine in response to stimulation with rF1-V and had higher LcrV antibody titers than animals receiving LMA vaccine first during the long-term study (Fig. 5E and 6). Therefore, in this regard, delivering the Ad5-YFV vaccine first followed by LMA vaccine would be preferred and is much more attractive based on safety, and both vaccines can be administered i.n., thus developing a more acceptable adjuvant- and needle-free administration protocol from the public health prospective.

The heterologous vaccination strategies have been previously used for diseases in which cell-mediated responses were particularly important for protection, such as in patients with HIV and malaria (52). Recently, during the COVID-19 pandemic, the heterologous prime-boost vaccination has been emphasized mainly due to the shortage of available COVID-19 vaccines (53). The intentional design has also been reported in the Russian Sputnik vaccines, which use two different adenovirus vectors (type 5 and type 26); thus far, this is the only heterologous prime-boost vaccine to be licensed for human use (54–56). Further, the many COVID-19 vaccines under development based on different platforms and strategies, and the likely need for additional boosters due to the emerging COVID-19 variants, have led to more enthusiasm in investigating the advantages of heterologous prime-boost approaches over a homologous boost strategy.

Indeed, it was shown that using a heterologous boost of either adenoviral-vectored vaccine or mRNA-based COVID-19 vaccines improved neutralizing antibody titers with induction of stronger Th1 responses than a homologous boost of inactivated SARS-CoV-2 vaccines (57). Similarly, using an mRNA-based COVID-19 vaccine as a booster (BNT162b2) in a heterologous approach instead of using a homologous adenoviral booster (ChAdOx1-nCov-19) resulted in increased neutralizing antibody titers and SARS-CoV-2-specific T cells (58). Although there are no data for direct comparison between heterologous and homologous immunization approaches with LMA and Ad5-YFV vaccines, we did notice that the antibody titers to the individual antigen LcrV were the lowest among three tested antigens (LcrV, F1, and YscF) in mice immunized solely with the Ad5-YFV vaccine (26). In contrast, when mice were immunized with both LMA and Ad5-YFV vaccines in the heterologous format, a comparable level of antibodies to all three antigens was observed (Fig. 5E).

Recently, a prime and pull immunization regimen has been investigated in a variety of vaccines with success (59–61), which indicates that the immune response at a mucosal site can be triggered by the administration of an antigen to a distant mucosal site. In this strategy, a parenteral vaccination raises systemic cellular response (prime) followed by a mucosal delivery of chemokine or vaccine that directs the tissue targeting of the prime-activated circulating T cells (pull) (62). In our study, mice i.m. immunized with LMA followed by i.n. delivery of Ad5-YFV vaccine exactly fit this category and should be further investigated. Although significant increases in splenic T-cell populations (both CD4 and CD8) was observed in all immunized mice compared to the naive controls, no significant differences among all of the immunized groups of mice was observed. In addition to the circulating T cells, the hallmark of prime-pull immunization is the elevated local T-cell population, especially the resident memory T cells (TRM), at the mucosal sites (59–61). TRM occupies tissues without recirculating, provides a first response against infections, and accelerates pathogen clearance (63). Therefore, it is possible that the local level (lungs) of T cells varies among our different heterologous immunized groups of animals and needs to be further studied.

A new vaccination strategy by combination of both simultaneous and prime-boost immunizations has been used for H1N1 signal minus influenza vaccine (S-FLU) in a pig model (64). During the prime-boost immunization course, the study has shown pigs only immunized i.m. generated a high titer of neutralizing antibodies but poor T-cell responses, whereas aerosol solely induced powerful respiratory tract T-cell responses but a low titer of antibodies. However, immunization of animals with S-FLU via both i.m. and aerosol routes simultaneously during the prime-boost immunization course generated high antibody titers and strong local T-cell responses with the most complete suppression of virus shedding and the greatest improvement in pathology (64). This strategy has been highly recommended for TB vaccines as well (51). Considering the Ad5-YFV (i.n.) and LMA (i.m.) simultaneous combination was the only one among all immunized groups that showed the immune characteristics of both Ad5-YFV (Th1) and LMA (Th17) (Fig. 4B and E), it will be exciting to carry out a similar experiment with LMA and Ad5-YFV vaccines in lieu of S-FLU in the future.

Finally, cytokine/chemokine production (e.g., IL-1α, IL-1β, and IL-6) from splenocytes of unvaccinated mice 72 h p.i. with CO92 indicated a highly inflammatory environment with neutrophil chemoattractants (CXCL1, RANTES, and G-CSF) at elevated levels (Fig. 11). None of these cytokines/chemokines were elevated in any of the combinations of heterologous prime-boost-vaccinated mice, indicating the inability of Y. pestis to cause immune dysregulation during early stages of infection. Conversely, immunized and infected mice in all heterologous prime-boost groups had higher levels of CCL2, and some groups had higher CCL4, which could be essential in recruiting monocytes to clear the invading pathogen. Importantly, mice immunized with the LMA vaccine first had the largest amount of secreted IL-17 during the postinfection time point, which, combined with decreases in CXCL1, RANTES, and G-CSF and increases in CCL2, CCL4, and GM-CSF, could have a critical role during productive versus nonproductive stages of pneumonic plague.

In general, by using a heterologous vaccination strategy, we have clearly demonstrated that the combination of vaccines, the route and timing of administration, and the length of the vaccination schedule are all crucial factors that affect efficiency and safety of vaccinations. It is important to reiterate that almost all our heterologous combinations offered complete protection from high-dose challenges of both CO92 and its CAF^−^ strain, and each one of them has its own characteristics that can fit different scenarios. Importantly, the 2-dose regimens, especially the Ad5-YFV (i.n.) and LMA (i.n.) combination, are ideal for the routine immunization in plague regions of endemicity, while the simultaneous approach with Ad5-YFV (i.n.) and LMA (i.m.) would be beneficial for vaccination in response to emergency situations.

Our future studies will address three important questions and include (i) comprehensively assessing potency of memory responses (T and B cells) that would guide us on dosing strategies; (ii) assessing antibody potency in neutralizing Y. pestis infection, which would also support our heterologous prime-boost approach with two vaccines and potentially reveal other additional differences that are important in humans; and (iii) testing a heterologous prime-boost strategy in a humanized mouse model and nonhuman primates to gauge the superiority of our approach compared to competing vaccine candidates and that our Ad5-YFV and LMA combination would be highly efficacious in humans.

## MATERIALS AND METHODS

### Bacterial strains and vaccines.

A fully virulent human pneumonic plague isolate, the parental Y. pestis strain (CO92), was obtained from BEI Resources (Manassas, VA). The F1-negative strain (CAF^−^) was created in our laboratory by deletion of partial *caf1A* and most of the *caf1* gene from CO92. The mutant strain retained its virulence in both pneumonic and bubonic plague animal models (39). The live attenuated vaccine candidate (LMA) is a triple deletion mutant of CO92 in which genes encoding Lpp, MsbB, and Ail were deleted (19). The KIM/D27 strain of Y. pestis deleted for the pigmentation locus required for iron acquisition from the host was used in an iron overload experiment performed in mice (65). The Ad5-YFV is a human replication-defective adenovirus type 5 vector-based vaccine containing genes for three plague antigens: F1, LcrV, and YscF (25). All studies involving Y. pestis were performed in Tier 1 select agent laboratories at UTMB in the Galveston National Laboratory (GNL), Galveston, TX.

A large batch of Ad5-YFV vaccine (1 × 10^16^ virus particles [v.p.]/batch, aliquoted in 1 ml at 1 × 10^12^ v.p. and stored at −80°C) was prepared from a 20-liter suspension culture of HEK293 cells in a chemically defined, protein-free CD-293 medium. The vaccine was purified at the Baylor College of Medicine Vector Development Laboratory and by our company partner in collaboration with Lonza, Houston, TX, under good laboratory practice conditions. This batch of vaccine was used for our subsequent studies in mice and nonhuman primates (25, 26). Likewise, a large batch of the LMA vaccine (2 liters) was prepared under a highly regulated quality control system in a GNL biosafety level 3 (BSL-3) suite by growing in heart infusion broth (HIB) overnight at 28°C as a shake flask culture (180 rpm). The culture was centrifuged, washed with HIB, and resuspended to 1/20 the original volume. The culture was aliquoted (500 μL, ∼1 × 10^9^ CFU/ml) with 25% glycerol and stored at −80°C. Titers of the vaccines were confirmed before and after each inoculation, and the same batches of the vaccines were used throughout our earlier and these studies (19, 27).

### Animals.

Outbred Swiss-Webster (female) and inbred C57BL6 Rag1 knockout (KO; lacking mature T and B cells, male and female) mice (6 to 8 weeks) were purchased from the Jackson Laboratory (Bar Harbor, ME). All experiments were conducted in the animal biosafety level 3 (ABSL-3) facility at UTMB in the GNL. Studies were ethically performed under an approved Institutional Animal Care and Use Committee protocol.

### Iron overload studies.

Swiss-Webster mice (*n* = 5/group) were injected with 75 μg of ferrous chloride (FeCl_2_·4H_2_O; Sigma-Aldrich Inc., St. Louis, MO) by the intraperitoneal (i.p.) route (65) and then challenged i.n. with either the KIM/D27 strain or the LMA vaccine strain (2 × 10^5^ to 5 × 10^6^ CFU/50 μl). A similar number of untreated mice (without FeCl_2_·4H_2_O) were also infected and served as controls. The animals were observed for body weight loss, other clinical signs of disease (ruffled fur, hunched back, lethargy, lack of grooming, sunken eyes, squinting of eyes with ocular and/or nasal discharge, open-mouth breathing, and gasping for air), and morbidity over a period of 14 days.

### Rag1 KO mouse studies.

Rag1 KO mice (*n* = 10, males or females) were infected with 4 LD_50_ of CO92 by either the i.n. or i.m. route and observed for morbidity and mortality. For inbred mice, 1 LD_50_ equaled 10 or 100 CFU when delivered by the i.m. or the i.n. route, respectively. Lungs, liver, and spleen were excised from moribund mice and homogenized in 1 to 2 ml of phosphate-buffered saline (PBS). Homogenates were 10-fold serially diluted and plated on sheep blood agar (SBA) plates to quantify bacterial load. For the LMA vaccine studies, Rag1 KO mice (10 for each infection route) were infected with 2.0 × 10^6^ CFU by either the i.n. or the i.m. route. A cohort of 10 mice (5 from each infection route) was sacrificed on day 5 postinfection (p.i.), and bacterial loads in spleen and at the initial infection sites (lungs or the muscle) were determined as described above. The remaining 10 mice were observed for signs of disease for 28 days, and the survivors were then i.n. challenged with 6 LD_50_ of CO92. On day 3 postchallenge, mice were bled to collect serum, and lungs from 5 moribund animals were excised on day 4 to quantify bacterial load.

### Heterologous vaccination and challenge studies.

Swiss-Webster mice were immunized with Ad5-YFV and LMA vaccines in either a 1- or 2-dose regimen. In a 1-dose regimen, both Ad5-YFV and LMA were delivered simultaneously, while in a 2-dose regimen, Ad5-YFV and LMA vaccines were administered 21 days apart in various orders and route combinations. Mice receiving PBS were used as controls. The vaccination doses were 1.2 × 10^10^ PFU/40 μl for Ad5-YFV and 2.0 × 10^6^ CFU/50 μl for the LMA vaccine (26, 27).

For short-term studies, the immunized and naive control mice (*n* = 8 to 10) were retro-orbitally bled on day 21 after the last dose of immunization. The animals were then i.n. challenged with 100 LD_50_ of CO92 or CAF^−^ mutant on day 24 after completion of the vaccination course. For Swiss-Webster mice, 1 LD_50_ equaled 500 CFU of CO92 or CAF^−^ by the i.n. route (21, 39). In a separate experiment, mice (*n* = 5) were similarly immunized with various combinations as described above; however, the group in which LMA and Ad5-YFV vaccines were administered simultaneously via the i.n. route was excluded because of interference in generating immune responses and reduced animal protection after bacterial challenge (see Results). On day 21 after completion of the immunization course, mice were euthanized and spleens collected for the evaluation of cell-mediated immunity.

For long-term studies, mice were immunized with the 2-dose regimen only, and animals receiving PBS were used as controls. Blood and spleens were collected from 5 mice in each immunized and control group on day 63 (42 days after last immunization dose), and the rest of mice were challenged with 100 LD_50_ of either CO92 or its CAF^−^ strain on day 105 (84 days after last immunization dose). On day 3 postchallenge with CO92, organs (spleen, lungs, and liver) were collected from all moribund animals to quantify bacterial load, and spleens were also excised from 5 live animals (after euthanization) of each group. At the end of the experiment, bronchoalveolar lavage fluid (BALF), spleen, and lungs or muscle were collected from all the surviving animals. The isolated sera and BALFs were filtered using 0.1-μm filter cartridges (Millipore Sigma Life Science Center, Burlington, MA) and sterility confirmed before performing subsequent experiments at a lower biocontainment level. The collected organs were used for quantitation of the bacterial load or for the evaluation of cell-mediated immunity.

### Antibody titer analysis.

Antibody titers were measured by performing indirect enzyme-linked immunosorbent assay (ELISA) (26). Briefly, MaxiSorp ELISA plates (Nunc, Rochester, NY) were coated with 100 ng of recombinant fusion protein (rF1-V) (BEI Resources) or individual plague antigen rF1, rLcrV, or rYscF in carbonate buffer (100 μl) at 4°C overnight. Noncoated antigens were removed with 3 washes of Dulbecco’s PBS (DPBS) with 0.05% Tween 20. Plates were then blocked with 1% powdered milk (EMD Chemicals Inc., Gibbstown, NJ) in DPBS for 1 h at room temperature. After 3 more washes, sera or BALFs were 2-fold serially diluted and incubated for 1 h at room temperature. Plates were again washed 3 times, and then horseradish peroxidase (HRP)-conjugated secondary anti-mouse antibodies for IgG, IgG1, IgG2a, or IgA (Southern Biotech, Birmingham, AL), diluted at 1:8,000, were added and incubated for 1 h at room temperature. Plates were washed 3 times, and then 100 μl of TMB (3,3′,5,5′-tetramethylbenzidine) substrate was added for 5 to 15 min at room temperature. Colorimetric reaction development was stopped using 2N H_2_SO_4_. Absorbance was then measured at 450 nm using a VersaMax tunable microplate reader (Molecular Devices San Jose, CA).

### T-cell phenotypes.

Spleens collected from both immunized and control mice were smashed and passed through a 70-μm cell strainer to obtain a single-cell suspension in RPMI 1640 cell culture medium. Splenocytes were then seeded into 24-well tissue culture plates at a density of 2.0 × 10^6^ cells/well. Four wells/mouse/plate were treated with ionomycin (750 ng/ml, calcium ionophore), PMA (phorbol 12-myristate 13-acetate; protein kinase C activator, 50 ng/ml), and brefeldin A (5 μg/ml) for 5 h at 37°C in a 5% CO_2_ incubator. Stimulation of splenocytes with PMA and ionomycin leads to activation of several intracellular signaling pathways, bypassing the T-cell membrane receptor complex, resulting in strong T-cell activation and production of a variety of cytokines. Splenocytes were then blocked with anti-mouse CD16/32 antibodies (BioLegend, San Diego, CA) followed by staining with fixable viability dye eFluor 506 (eBioscience, San Diego, CA) and allophycocyanin anti-mouse CD3e (eBioscience), phycoerythrin (PE)/Dazzle 594 anti-mouse CD4 (BioLegend), and fluorescein isothiocyanate (FITC) anti-mouse CD8 (BioLegend) for CD3, CD4, and CD8 T-cell surface markers, respectively. Cells were then permeabilized for intracellular staining with peridinin chlorophyll protein/Cy5.5 anti-mouse gamma interferon, PE/Cy7 anti-mouse IL-17A (BioLegend), and analyzed by flow cytometry.

### Cell proliferation and cytokine production.

To measure T- and B-cell proliferation, bromodeoxyuridine (BrdU), a thymidine analog, incorporation method was used. Briefly, the isolated splenocytes were seeded in duplicate 24-well tissue culture plates (1.0 × 10^6^ cells/well) and stimulated with rF1-V fusion protein (100 μg/ml) for 72 h at 37°C. BrdU (BD Bioscience, San Jose, CA) was then added into the wells of one of the plates at a final concentration of 10 μM during the last 18 h of incubation, with rF1-V to be incorporated into newly synthesized DNA of the splenocytes (66, 67).

Subsequently, the BrdU-labeled splenocytes were surface stained for T-cell (CD3e-APC; eBioscience) and B-cell (CD19-eFluor450; ThermoFisher Scientific, Grand Island, NY) markers after blocking with anti-mouse CD16/32 antibodies (BioLegend). Cells were then permeabilized and treated with DNase to expose BrdU epitopes, followed by anti-BrdU-FITC and 7-AAD (7-amino-actinomycin D) staining by using a BD Pharmingen FITC BrdU flow kit (San Jose, CA). The splenocytes were then subjected to flow cytometry and data analyzed as we previously described (28). The percentages of BrdU-positive cells in CD3- and CD19-positive populations were calculated using FACSDiva software.

To assess cytokine production, cell supernatants were collected from a duplicate plate after stimulation with rF1-V (100 μg/ml) for 72 h at 37°C. For these studies, we used Y. pestis-specific antigens for stimulation to confirm flow cytometry data. Cytokines in the supernatants were then measured by using Bio-Plex Pro mouse cytokine 23-plex assay (Bio-Rad Laboratories, Hercules, CA) by following the manufacturer’s standard protocol.

### Statistical analysis.

One-way or two-way analysis of variance (ANOVA) with Tukey’s *post hoc* test or the Student's *t* test was used for data analysis. We used Kaplan-Meier with log-rank (Mantel-Cox) test for animal studies, and *P* values of ≤0.05 were considered significant for all the statistical tests used. The number of animals per group is described in each figure or figure legend, and two biological replicates were performed. All *in vitro* studies were performed in triplicates.
