# Supplementary material for: Combinatorial Viral Vector-Based and Live Attenuated Vaccines without an Adjuvant to Generate Broader Immune Responses to Effectively Combat Pneumonic Plague
Source: mBio. 2021 Dec 7;12(6):e03223-21. doi: 10.1128/mBio.03223-21 (PMC8649767; doi:10.1128/mBio.03223-21)
Supplement: FIG S1 [file mbio.03223-21-sf001.pdf]

Fig. S1

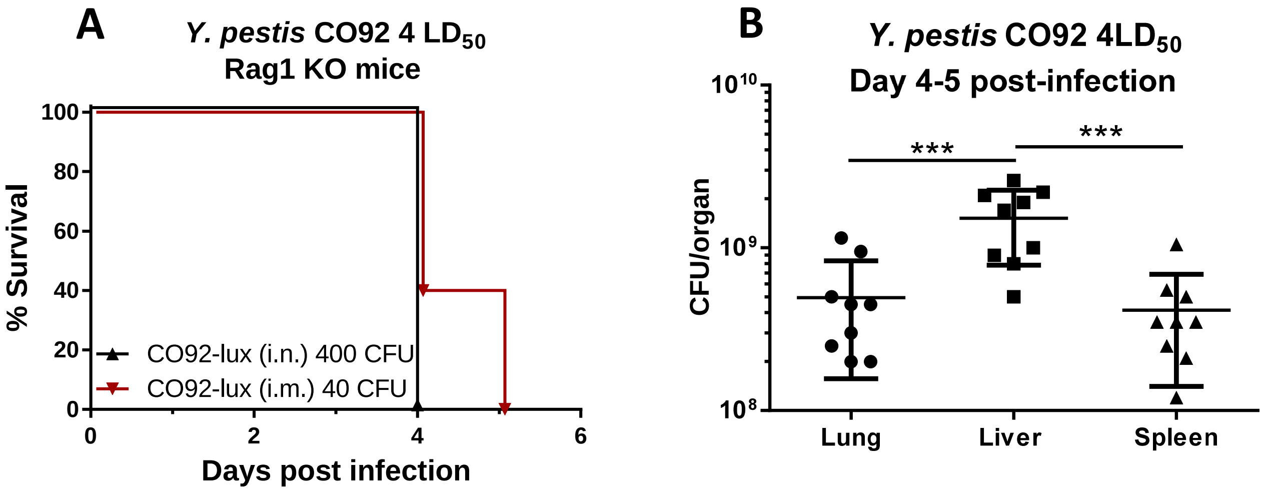

Figure S1. Parental *Y. pestis* CO92 causes clinical disease in Rag1 KO mice. C57BL6 Rag1 KO mice (n=4-5 each route) were infected with 4 LD_{50} of CO92 by either (i.n.) or (i.m.) route and the mortality of animals recorded and plotted (**A**). Lungs, liver, and spleen were removed from the moribund animals to quantify the bacterial loads (**B**). Kaplan-Meier analysis with log-rank (Mantel-Cox) test was used for analysis of animal survival (**A**). Asterisks represent the statistical significance between two groups indicated by a line and *** $p<0.001$ (**B**). Two biological replicates were performed, and data plotted.
