# Supplementary material for: Combinatorial Viral Vector-Based and Live Attenuated Vaccines without an Adjuvant to Generate Broader Immune Responses to Effectively Combat Pneumonic Plague
Source: mBio. 2021 Dec 7;12(6):e03223-21. doi: 10.1128/mBio.03223-21 (PMC8649767; doi:10.1128/mBio.03223-21)
Supplement: FIG S2 [file mbio.03223-21-sf002.pdf]

Fig. S2

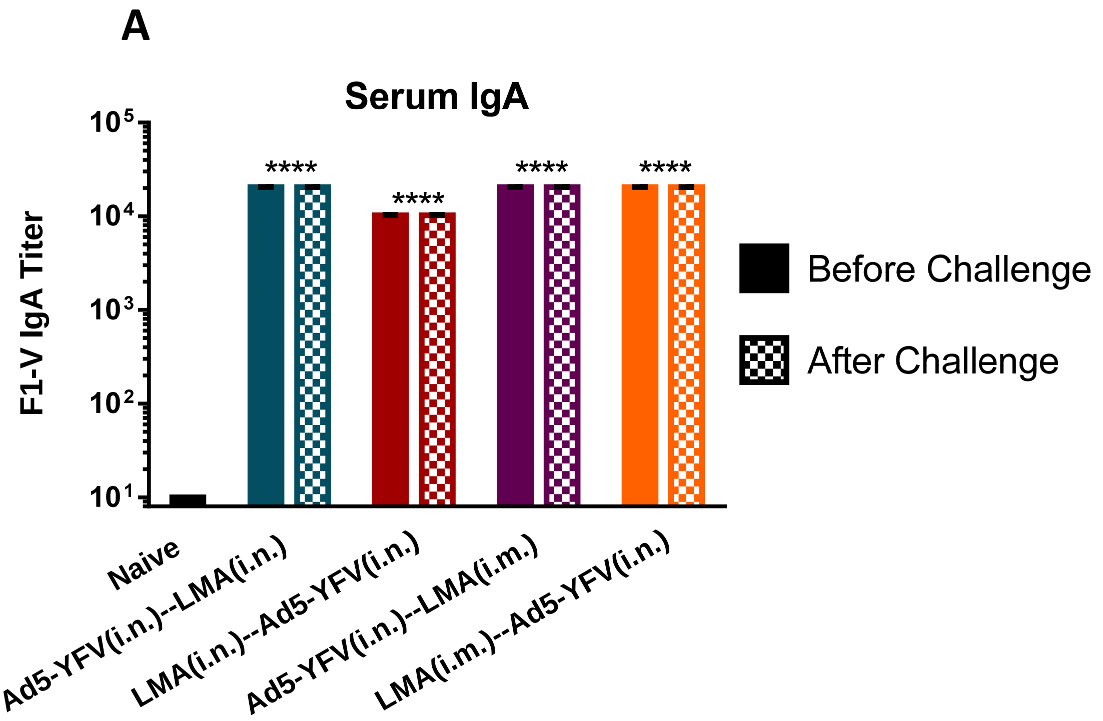

Figure S2. No significant differences in F1-V specific serum IgA are observed in vaccinated mice before and after infection. Mice were immunized with Ad5-YFV and LMA vaccines in 2-dose (prime-boost) regimens in which Ad5-YFV and LMA were administered 21 days apart in various combinations (Fig 5A). Serum was collected 42 days after the 2nd vaccination as well as 28 days post-infection. F1-V specific IgA was determined by ELISA. Titers were determined in triplicate. One-way ANOVA with Tukey's post hoc test was used to determine significant differences between groups. Asterisks indicate significance compared to naïve serum. **** p<0.0001.
